# PEG Conjugated Zein Nanoparticles for In Vivo Use

**DOI:** 10.3390/pharmaceutics14091831

**Published:** 2022-08-31

**Authors:** Courtney van Ballegooie, Nicole Wretham, Tanya Ren, Ioana-Mihaela Popescu, Donald T. Yapp, Marcel B. Bally

**Affiliations:** 1 Experimental Therapeutics, BC Cancer Research Institute, Vancouver, BC V5Z 4E6, Canada; 2 Faculty of Medicine, University of British Columbia, Vancouver, BC V6T 1Z3, Canada; 3 NanoMedicines Innovation Network, Vancouver, BC V6T 1Z3, Canada; 4 Faculty of Pharmaceutical Sciences, University of British Columbia, Vancouver, BC V6T 1Z3, Canada

**Keywords:** polyethene glycol, nanoparticles, Zein, protein-based drug delivery systems, nanomedicine, microfluidics

## Abstract

Zein can be utilized to form nanoscale particles for drug delivery applications. Despite the ease of synthesis, these particles often aggregate when exposed to physiologically relevant conditions (e.g., pH and salt concentrations). This instability has prevented their further development in applications requiring intravenous administration. To mitigate this colloidal instability, this research explored Zein nanoparticles (NP)s that were modified with polyethylene glycol (PEG) either through functionalized PEG pre- or post-NP formation. The results suggest that the pre-functionalization of the Zein using N-hydroxysuccinimide ester terminated PEG is the method of choice for synthesizing Zein NPs with conjugated PEG (Zein:PEG-Zein NPs). Zein:PEG-Zein NPs formed using this method displayed excellent stability in physiologically relevant conditions over 72 h and were stable at 4 °C for at least 3 months. When the NPs were cultured with cells for 72 h, no cytotoxicity or early signs of apoptosis were identified. Cellular uptake of the Zein:PEG-Zein NPs did not seem to be impacted by the amount of PEG incorporated in the NP but were concentration-, time-, and temperature-dependent. The lowest percent, stable Zein:PEG-Zein NP formulation (80% unmodified Zein and 20% PEG-modified Zein) induced no observable toxicity over 14 days in CD-1 mice dosed at 70 mg/kg via the tail vein. However, repeat dose pharmacokinetic (PK) studies demonstrated that following the first dose, the second dose caused health issues that required euthanasia shortly after administration. For those animals that survived, there was faster plasma elimination of the Zein:PEG-Zein NPs. Despite this, the Zein:PEG-Zein NPs represent a significantly improved formulation approach, one that displays a long circulation half-life and is suitable for single-use administration. Repeat dose applications will require additional methods to silence the immune response that is generated when using these NPs intravenously.

## 1. Introduction

Nanoparticle-based drug delivery systems have been investigated to modulate the characteristics of small molecule drugs, impacting such features as their efficacy, bioavailability and circulation half-life. Nanoparticles (NP)s can also play an important role in stabilizing drugs which exhibit limited solubility in aqueous solutions [1]. In therapeutic applications for cancer, NPs can localize at the tumor site due to leaky tumor vasculature in a process known as the enhanced permeability and retention (EPR) effect [2]. This, in turn, increases the accumulation of the NPs and, by extension, the amount of loaded drug at the target site; the resulting benefit would be the increase in the drug′s efficacy with a corresponding decrease in the drug’s side effects [1,2]. While there have been reports indicating that tumor heterogeneity impacts the EPR effect, and the significance of the EPR effect has been brought to question, there is no question that appropriately designed NPs will be retained in the plasma compartment over extended time periods and ultimately cause prolonged exposure of the tumor to the drug [3].

Zein, a prolamin protein extracted from corn, is an attractive component for preparing NP candidates. The literature suggests that this protein (1) is biocompatible, is formulated in non-toxic conditions, and has been given a generally recognized as safe status by the Food and Drug Administration (FDA) for topical use as a biodegradable patch and oral use as a tablet coating; (2) is derived from the endosperm of corn and is, therefore, a renewable and abundant resource; (3) contains a large number of reactive moieties available for functionalization; (4) is amphiphilic and hydrophobic in nature and possesses the ability to form NP structures without the need of additional chemical modification [4]. While Zein NP formulations have not reached the stage of clinic utility, Ethibloc^®^, a solubilized formulation of Zein, is clinically approved within Canada and has been investigated for the treatment of epistaxis, aneurysmal bone cysts, and lymphangiomas [5,6]. Composed of a high concentration of Zein in an alcoholic solution, Ethibloc is a viscous substance that progressively hardens in a wet milieu, thereby allowing the local occlusion of blood vessels when injected. In long-term studies, Ethibloc was found to be a safe and effective treatment for lymphangiomas and aneurysmal bone cysts with minor short-term complications, commonly described as “flu-like” symptoms, and no long-term side effects have been noted when injected in single- or multi-dose schema [7,8,9]. Although Ethibloc is a non-NP formulation of Zein, it is nonetheless highly encouraging that this protein appears to be biocompatible and useful within a clinical setting. The research described herein aims to develop a Zein-based NP formula for parenteral applications.

**Figure 1:**
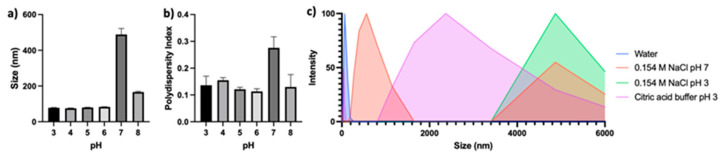
Stability of Zein nanoparticles (Zein NPs; 70 +/− 10 nm upon synthesis) in varying conditions: (**a**) Size of Zein NPs at a pH ranging from three to eight unbuffered and adjusted directly with HCl or NaOH; (**b**) polydispersity of Zein NPs at a pH ranging from three to eight unbuffered and adjusted directly with HCl or NaOH; and (**c**) intensity histogram of Zein NP size in water, 0.145 M NaCl at pH 7, 0.145 M NaCl at pH 3 unbuffered and adjusted directly with HCl, and citric acid buffer at pH 3.

While many synthesis methodologies and encapsulation techniques have been investigated for Zein NPs, considerable challenges remain because of the colloidal stability of the Zein NPs. Specifically, Zein NPs have been shown to aggregate and precipitate near or above the protein’s isoelectric point of 6.8 (Figure 1a,b) [10,11]. Similarly, the addition of low concentrations of salt have been shown to induce aggregation and precipitation as well (Figure 1c) [12,13]. Salt addition increases the ionic strength of the solution, and this results in increased van der Waals interactions and hydrophobic effects among the protein chains. This promotes the aggregation of the NPs and precipitation of the proteins [13,14]. To remedy this stability challenge, investigators have considered a range of ideas, including thermal treatment of the proteins and the use of coating agents and emulsifiers, such as carrageenan, gum arabic, lecithin, Pluronic^®^, sodium caseinate, pectin, and chitosan. Despite these strategies, many of the NP formulations exhibit poor stability properties or have not undergone further characterization in salt-containing or neutral pH environments [13].

**Figure 2:**
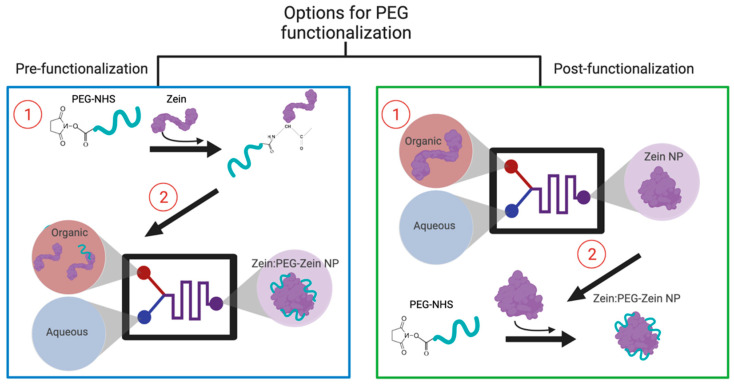
Illustration of the polyethylene glycol (PEG) conjugation methods (pre- and post-functionalization of Zein and Zein nanoparticles (NP)s, respectively) that could be utilized for PEGylation of Zein via a PEG-N-hydroxysuccinimide (NHS) ester reaction.

Polyethylene glycol (PEG) is a hydrophilic, highly water-soluble, putatively non-immunogenic, and non-toxic polymer suitable for bioconjugation. PEG is currently approved by the FDA for drug delivery applications, and its use has led to the successful application of lipid-based NP formulations (e.g., Doxil^®^ and Onivyde^®^) in the clinic. Varieties of different NPs have been PEG-modified, including dendrimers, liposomes, metallic NPs, and synthetic polymers. It has been argued that PEG-modified NPs display favorable physiochemical behavior, improved biocompatibility, and enhanced serum stability. It has been suggested that strategies to surface modify NPs with PEG can lead to “stealth” behavior in vivo, but this is dependent on a variety of factors, including the encapsulated material [15,16]. As noted by Dos Santos et al., the primary role of PEG is to prevent surface–surface interactions that can lead to particle aggregation [17]. Despite the potential of using PEG to stabilize Zein NPs, there are no published studies characterizing how best to prepare PEG conjugated Zein NPs. As noted in Figure 2, the research here explored pre- and post-PEG functionalization strategies for Zein NPs. The functionalization strategies explored herein utilize a PEG-N-hydroxysuccinimide (NHS) ester, which reacts readily with available primary amines, such as those found within the N-terminus of Zein. Perhaps not surprisingly, the attachment of PEG to preformed Zein NPs was problematic, as the chemical reaction required the use of salts and neutral pHs, which led to Zein NP aggregation. Modification of the Zein protein, prior to Zein NP formation, was successful, stabilizing the NPs for use in salts and in serum; this allowed an assessment of the pharmacokinetic (PK) characteristics of the Zein NPs.

## 2. Materials and Methods

### 2.1. Materials

Zein from maize (Cat. 9010-66-6) was purchased from Sigma-Aldrich as well as a 40% acrylamide/bis-acrylamide solution (Cat. A7802) acetic acid (Cat. 695092), hydrochloric acid (Cat. 258148) and 2-mercaptoethanol (Cat. M3148). DMSO (Cat. D2438), Poloxamer 188 solution (Cat. P5556), Tween 80 (Cat. P4780), and fluorescamine (Cat. F9015) were purchased from Sigma. The following items were purchased from Bio-Rad Laboratories: TEMED (Cat. 161-0800), ammonium persulfate (Cat. 161-0700), SDS (Cat. 161-0302), resolving buffer (Cat. 161-0798), stacking buffer (Cat. 161-0799), 10× Tris/Glycine buffer (Cat. 161-0734), 4× Laemmli Sample Buffer (Cat. 161-0747). A Pierce Silver Stain Kit (Cat. 24612) and bicinchoninic acid assay (BCA) kit (Cat. 23227) were purchased from ThermoFisher. Ethyl alcohol anhydrous was purchased from Commercial Alcohols (Cat. P016EAAN). Sterile Acrodisc^®^ syringe filters with Supor^®^ membrane (13 mm, 0.80, 0.45, and 0.22 µm membrane; Cat. 4608, 4604, and 4602, respectively) and Acrodisc syringe filters with Tuffryn^®^ membrane (0.22 µm membrane; Cat. 4454) were purchased from Pall. NALGENE 0.45 and 0.22 µm cellulose membrane syringe filters (Cat. 190-2545 and 190-9920 respectively) were purchased from Voigt Global Distribution Inc. 5 kDa PEG-N-hydroxysuccinimide ester (Cat. PG1-SC-5k-2) was purchased from Nanocs. The 500 kD tangential flow filtration membrane (Cat. C02-S500-05-N) was purchased from Repligen Corporation. Slip tip syringes for 3 mL (Cat. 309586) and 1 mL (Cat. 309659) volumes were purchased from Becton Dickinson. The 384-well flat bottom black polystyrene TC-treated microplates were purchased from Corning (Cat. 3764). A protein ladder (Cat. PM007-0500S) was purchased from FroggaBio Scientific Solutions. RPMI-1640 media (Cat. 11875-093), DMEM (Cat. 11995-065), FluoroBrite^®^ DMEM (Cat. A18967-01), DMEM F-12 (Cat. 11320-033), fetal bovine serum (Cat. 12483-020), 0.25% trypsin-EDTA (Cat. 25200-056), L-glutamine (Cat. 25030149), 10% Pluronic^®^ F-68 (Cat. 24040-032), Hoechst 3342 trihydrochloride (H3570), CellEvent™ Caspase-3/7 Green Detection Reagent (C10723), and BackDrop Background Suppressor ReadyProbes Reagent (Cat. R37603) were purchased from Life Technologies (Carlsbad, CA). McCoy 5A media (Cat. 36350) was purchased from StemCell Technologies. Sephadex^®^ G-50 Medium (17-0043-01) was purchased from GE Healthcare. Ethidium homodimer I (Cat 40010) was purchased from Biotium. Zein NPs were synthesized using a Benchtop NanoAssemblr (Precision Nanosystems). Samples were analyzed using a NanoBrook ZetaPALS for DLS. Ultraviolet Spectroscopy (UV-Vis) was performed on a Nanodrop Spectrophotometer ND-1000 and a Mandel CLARIOstar microplate reader from BMG LABTECH. Samples were shaken using a Thermomixer R from Eppendorf unless otherwise specified. Centrifugation was performed on an Thermoscientific Legend Micro 21R Centrifuge using Eppendorf tubes (VWR, Cat 20170-577) unless otherwise specified. Samples were heated using an Accublock Digital Dry Bath from Labnet International. Sonication was performed on a 2510 Ultrasonic Cleaner from Branson (40 kHz frequency). Gels were run on a Bio-Rad Mini-PROTEAN^®^Tetra Cell and imaged using a Bio-Rad ChemiDoc MP Imaging system. Electron microscopy (EM) techniques were performed using a FEI Tecnai Osiris S/TEM for all TEM and STEM samples, while SEM images were acquired using a Nova NanoSEM with all samples being prepared on a holey 300-mesh formvar/carbon-coated copper grid (Cat. FCF-300; supplier Electron Microscopy Sciences, Hatfield, PA, USA). Cryogenic EM (Cryo-EM) samples were prepared using a Vitrobot from ThermoFisher.

### 2.2. Zein Purification

Zein was purified before use as previously reported in our lab [18]. Briefly, Zein (0.10 g) was suspended in 15 mL of anhydrous EtOH [18]. The protein suspension was then stirred overnight (400 rpm at 4 °C). The insoluble Zein was then allowed to settle, and the supernatant was removed. The Zein was then resuspended in anhydrous EtOH. This process was repeated for a total of two times with the final suspension of the Zein being in 60% EtOH. This purified Zein was filtered using a 0.8 µm syringe filter and was then ready for use in further experiments. All Zein stocks were kept at 4 °C and used within 2 weeks from their final suspension.

### 2.3. Zein-PEG Pre-Functionalization, NP Synthesis, and Product Purification

**Figure 3:**
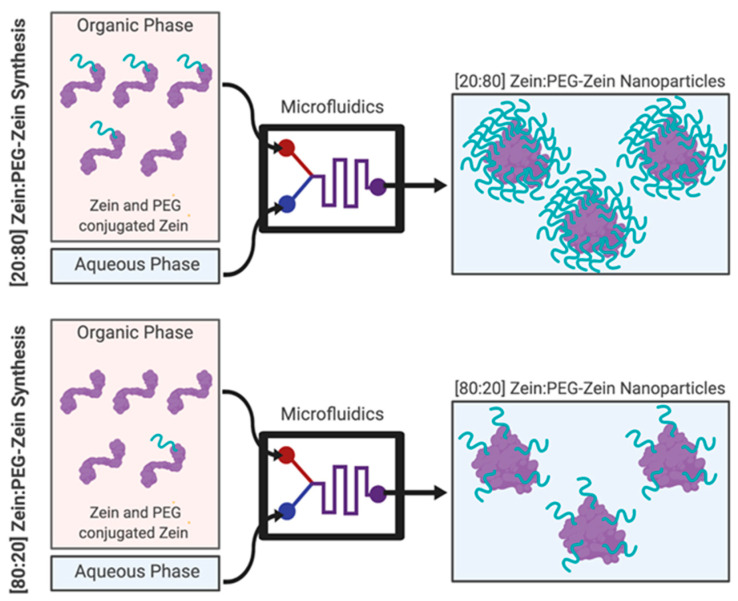
Illustration of the polyethylene glycol (PEG) pre-conjugation that could be utilized for the PEGylation of Zein via a PEG-N-hydroxysuccinimide (NHS) ester reaction. Example synthesis of the [20:80] and [80:20] Zein:PEG-Zein nanoparticle formulations depicted above.

The synthesis of PEG-conjugated Zein was adapted from a method published by Podaralla et al. [19]. 0.06 g of PEG-NHS ester was combined with 5 mL of a 1% (*w/v*) purified Zein solution (in 60% EtOH). The solution was covered and stirred for 24 h (4 °C) at 200 rpm. The 1% purified Zein was then combined at different ratios with the PEG-Zein before being subjected to the NP synthesis method using the microfluidics described previously in our lab [18]. Briefly, the various Zein:PEG-Zein ratios (Figure 3) were placed into a syringe (1.0 mL) as the organic phase in the first inlet channel in the microfluidics device. Its counterpart, the aqueous phase that contained deionized (DI) water (18.0 MΩ ultrapure water), was in a 3.0 mL syringe in the second inlet channel. The sample was run at a total flow rate of 2 mL/min and a relative flow rate of 3:1 (aqueous to organic). The samples were then collected at the microfluidics chip’s outlet channel. Each run was programmed to discard the first 0.3 mL of the sample and the last 0.1 mL of the sample. This ensured that the variability in fluid mixing dynamics that can potentially occur at the start and end of the synthesis run did not affect the NP formation. Samples synthesized with PEG-Zein were passed through a Sephadex G-50 spin column to separate the NPs from unassociated or loosely associated alcohol and/or dye. These samples were then subjected to tangential flow filtration (TFF) using a 500 kD cut-off membrane to help concentrate the sample. EtOH removal was confirmed using alcohol test strips. Samples then had their protein concentrations determined using UV-Vis, weigh (lyophilization), and/or BCA before further characterization. Further characterization, such as PEGylation verification, size, polydispersity (PD), and ζ potential, were then carried out.

### 2.4. Zein-PEG Post-Functionalization

Zein NPs without pre-functionalized PEG were synthesized using the microfluidics as described in Section 2.3. Samples were then stirred at 200 rpm for 24 h at room temperature to remove excess EtOH. EtOH removal was confirmed using alcohol test strips. Samples were then diluted to a final Zein concentration of 2.5 mg/mL. The following test conditions were investigated with all other parameters kept constant: the NHS ester concentration (0.1, 1, 2, 3, and 4 mg/mL), pH (3, 4, 5, 6, 7, and 8), acid used for pH modulation (acetic acid or HCL), presence of solvents (DMSO or EtOH), temperature (room temperature (~20 °C) and 4 °C), and presence of stabilizers (Pluronic and Tween 80). Samples were visually inspected for flocculation and analyzed by DLS.

### 2.5. SDS-PAGE

A 15% acrylamide running gel with 5% stacking gel was prepared for SDS-PAGE. The Zein and PEG-Zein samples (30 µL) were combined with a 3:1 Laemmli Sample Buffer containing BME (10 µL) and warmed at 100 °C for 10 min. Then, 5 µL of the protein ladder and 30 µL of the samples were loaded into each of the wells. A Glycine-Tris buffer with 0.1% SDS was used as the running buffer, and the samples were run at 200 V for 40 min. Gels were then stained using a Pierce Silver Stain Kit according to the manufacturer’s instructions. After staining, gels were imaged under a transilluminator (0.3 ms exposure) for further analysis.

### 2.6. Fluorescamine Assay

The 200 µg/mL of Zein and PEG-Zein were made from the stock using 60% EtOH as the diluent. After dilution, 150 µL aliquots of samples were pipetted into 96-well microplates. The microplate was placed on a microplate shaker, and 50 µL of a 10.8 mM (3 mg/mL) fluorescamine solution dissolved in acetone was added to each well. Following the addition of fluorescamine, the plate was shaken for 1 min at 100 rpm before the analysis. The fluorescence was then determined (400 nm, 30 nm band pass) with an excitation filter and a (460 nm, 40 nm band pass) emission filter; gain 860. A variation of this protocol was designed for Zein NPs and Zein:PEG-Zein NPs using 500 µg/mL of the samples with water as the dilutant. All other factors remained unchanged.

### 2.7. Nanoparticle Characterization and Stability Determination

Zein:PEG-Zein NPs were synthesized at the following ratios [100:0], [99:1], [95:5], [90:10], [80:20], [60:40], [40:60], [20:80], and [0:100] Zein:PEG-Zein using the microfluidics method described in Section 2.3. NP size and PD were assessed by diluting 10 µL of each sample in 3 mL of PBS, DMEM, or DI water. Samples were then allowed to sit for a minimum of 30 min at RT before being subjected to DLS. Samples were also exposed to 0.1 M KCl, and those that were stable in the salt solution were able to be further analyzed for ζ potential according to the manufacturer’s instructions. Samples that exhibited stability in the above conditions were then tested further for their stability in DMEM at 37 °C for up to 72 h, and their storage potential at 4 °C in water for up to three months.

### 2.8. Cell Culturing and Growth Rate Determination

A549 lung adenocarcinoma cells, 293t embryonic cells, Mia PaCa-2 pancreatic carcinoma cells, HDF cells, HT-29 colorectal adenocarcinoma cells, RAW 264.7 macrophage cells, J774A.1 macrophage cells, and MDA-MB-231 breast adenocarcinoma were purchased from ATCC (Manassas, VA, USA). A549 and MDA-MB-231 cell lines were cultured in RPMI 1640 medium, 293t, RAW 264.7, J774A.1, Mia PaCa-2, and HDF cell lines were cultured in DMEM medium, and HT-29 cells were cultured in McCoy 5A medium. All media were supplemented with 10% fetal bovine serum and 2 mM L-glutamine if instructed by the manufacturer. All in vitro experiments were performed between the third and eighteenth passages. All cell lines tested negative for mycoplasma. Cell doubling times were determined by plating 5000 cells/well of each cell line in triplicate for each future time point (24, 48, 72, and 96 h) on a 96-well plate. At the indicated time points, the cells were stained with Hoechst 33342 (2 µM) and ethidium homodimer (2 µM) for 20 min before being imaged using the InCell Analyzer 2000 under the DAPI and Cy3 filter sets.

### 2.9. Cell Toxicity

For the toxicity studies, cell lines were seeded in a 96-well plate as follows: A549 (3000 cells/well), 293t (3000 cells/well), Mia PaCa-2 (3000 cells/well), HDF (5000 cells/well), HT-29 (7000 cells/well), RAW 264.7 (3000 cells/well), BMDM (30,000 cells/well), MDA-MB-231 (7000 cells/well), J774A.1 (3000 cells/well) to account for their doubling times and surface area. Each cell line was seeded using 100 µL of appropriate culture media and allowed to adhere for 24 h overnight in an incubator at 37 °C under 5% CO_2_. Cells were then treated with 100 µL of the [20:80] or [80:20] Zein:PEG-Zein NP formulations at various final concentrations [0, 1, 10, and 100 µg/mL] diluted in culture media. At 24, 48, and 72 h after treatment, cells were washed three times with culture media before being stained with Hoechst 33342 (2 µM) and ethidium homodimer (2 µM) and incubated for 20 min in order to determine the total number of cells and dead cells, respectively. Cells were then imaged with the InCell Analyzer 2000 using the DAPI and Cy3 excitation and emission filter. Cell viability was calculated as described in Equation (1):

Cell viability (%) = ((Total cells − dead cells)/Total cells) × 100 (1)

At 72 h, an additional CellEvent Caspase 3/7 dye was utilized after the Hoechst and ethidium homodimer staining to determine the presence of early-stage apoptosis and imaged using the FITC filter. The final concentration of CellEvent was 5 µM, and the cells were allowed to incubate for 30 min before having their media replaced with FluoroBrite^®^ DMEM containing the green background suppressor as described by the manufacturer.

### 2.10. Cellular Uptake

Cell lines were seeded at 30,000 cells/well in a 96-well plate and allowed to adhere for 24 h overnight in an incubator at 37 °C under 5% CO_2_. Cells were then treated with 100 µL of a 1000 µg/mL Zein:PEG-Zein NP formulation coupled to CF-647, as described in Section 2.3. The variation to the protocol was made to incorporate CF-647 dye at a molar ratio of 1:0.002 Zein to dye by reacting the Zein to the dye in the same manner as the PEG-NHS ester. The formulation was then subjected to a Sephadex G-50 spin column using HBS as the exchange buffer and was sterile filtered. At fixed time points (e.g., 2, 4, 8, and 16 h), media containing the Zein:PEG-Zein NPs were removed, and the cells were washed three times before being stained with Hoechst 33342 (2 µM) for 20 min preceding imaging. Cells were then imaged with the InCell Analyzer 2000 using Bright Field, DAPI, and Cy5 excitation and emission filter.

### 2.11. In Vivo Safety and Pharmacokinetics

Preliminary safety studies were performed in CD-1 mice obtained from the Animal Resource Centre (ARC) at BC Cancer’s Vancouver Research Centre (Vancouver, BC, Canada), and single- and multi- dose PK studies were performed on Balb/CAnNHsd mice obtained from Envigo. All studies were conducted in accordance with the guidelines set by the Canadian Council on Animal Care, as overseen by the University of British Columbia’s Animal Care Committee. All mice were placed in Optimice cages (up to 4 mice per cage) and cages were equipped with nesting materials and plastic hiding structures (“huts”) for environmental enrichment. Animals were provided with food and water ad libitum. The health status of all animals used in these studies was monitored at least once daily following an established standard operating procedure designed to assess animal health status. Specifically, signs of ill health were based on body weight loss, change in appetite, and behavioral changes, such as altered gait, lethargy and gross manifestations of stress. If signs of severe toxicity were present, the animals were terminated (isoflurane overdose followed by CO_2_ asphyxiation) for humane reasons. At the end of the study, necropsies were performed to assess for overt signs of toxicity; none were noted.

Samples ([80:20] and [20:80] Zein:PEG-Zein NPs) were prepared as described in Section 2.3 with the CF-647-labeled format described in Section 2.10 for the single-dose PK study and the final dose in the multi-dose PK study. The animals were injected as a single-dose PK study or as part of a multi-dose study, where plasma levels were determined 4 h after administration. The formulations for injection were passed down an 80 mL Sephadex G-50 column and were then concentrated to 12 mg/mL via TFF with saline as the exchange buffer. The sample was then filter-sterilized through a sterile 5-mL 0.22 µm Suppor filter. The filter-sterilized formulation was then injected via the tail vein at a dose of 70 mg/kg (injection volume was 200 μL/20 g body weight), using a maximum volume of 300 µL. This was defined as the maximum feasible dose (MFD). A PEG-labeled liposomal (molar ratio of 50:45:5 DSPC:CHOL:PEG-DSPC) formulation containing DID and ^3^H was administered using a 27 mM lipid concentration, a dose of about 160 mg/mL to serve as a comparison (approximately 2 × 10^14^ NPs/mL) for clearance rates.

Following the IV administration of the [80:20] Zein:PEG-Zein NPs, CD-1 mice who were part of the safety study underwent clinical observation, as described above, and were weighed three times weekly for the duration of the study (14 days). Once the NPs safety was confirmed, the Balb/C mice who were a part of the single- and multi-dose study received either the [80:20] Zein:PEG-Zein NP, [20:80] Zein:PEG-Zein NP, or the PEG-labeled liposome formulation. Mice were terminated at 0.5, 1, 4, 8, and 24 h for the single-dose PK, and their blood was collected via a cardiac puncture and placed into EDTA tubes. Plasma was subsequently isolated by centrifugation (2000× *g* for 15 min at 4 °C) and then analyzed either via a scintillation counter or plate reader. Post-necropsy, organs (heart, kidney, lungs, liver, spleen, and lymph nodes) were harvested and imaged using the IVIS Lumina S5 with an excitation filter (640 nm; 20 nm bp) and emission filter (670 nm; 40 nm bp) for the 4 and 24 h single-dose PK and the 4 h assessment following multiple doses.

### 2.12. Statistics and Image Processing

Data were reported as the mean with the error bars representing the standard deviation (SD). Data sets had their normality tested using a Shapiro–Wilk test and/or a quantile–quantile plot when small sample sizes were present (*n* = 3). Equal variance was tested using a Brown–Forsythe test. Normally distributed data with equal variance then underwent an analysis of variance (ANOVA) or *t*-test. If the data were not normally distributed, then a non-parametric Kruskal–Wallis test, was employed. Multiple comparison tests, such as Tukey’s tests, were utilized to identify differences between sample means. Statistics were analyzed using Graph Pad Prism 9. Statistical significance was declared at the following probability levels: * *p* < 0.05, ** *p* < 0.01, ***p < 0.001, and ****p < 0.0001. Image processing to determine the integrated density of SDS-PAGE gels and NP uptake images utilized ImageJ.

## 3. Results

### 3.1. Zein-PEG Post-Functionalization

**Figure 4:**
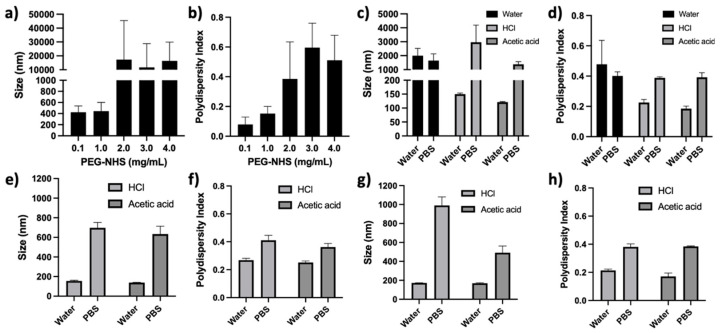
Post-functionalization of Zein nanoparticles (NPs) in varying experimental conditions: (**a**) size of Zein NPs at a PEG-N-hydroxysuccinimide (NHS) concentration between 0.1 and 4.0 mg/mL; (**b**) polydispersity of Zein NPs at a PEG-NHS concentration between 0.1 and 4.0 mg/mL; (**c**) size of Zein NPs post functionalized with 3.0 mg/mL PEG-NHS in an unbuffered system adjusted directly with HCl or acetic acid; (**d**) polydispersity of Zein NPs post-functionalized with 3.0 mg/mL PEG-NHS in an unbuffered system adjusted directly with HCl or acetic acid; (**e**) size of Zein NPs post-functionalized in 5% DMSO with 3.0 mg/mL PEG-NHS in an unbuffered system adjusted directly with HCl or acetic acid; (**f**) polydispersity of Zein NPs post-functionalized in 5% DMSO with 3.0 mg/mL PEG-NHS in an unbuffered system adjusted directly with HCl or acetic acid; (**g**) size of Zein NPs post-functionalized in 10% EtOH with 3.0 mg/mL PEG-NHS in an unbuffered system adjusted directly with HCl or acetic acid; and (**h**) polydispersity of Zein NPs post-functionalized in 10% EtOH with 3.0 mg/mL PEG-NHS in an unbuffered system adjusted directly with HCl or acetic acid.

**Figure 5:**
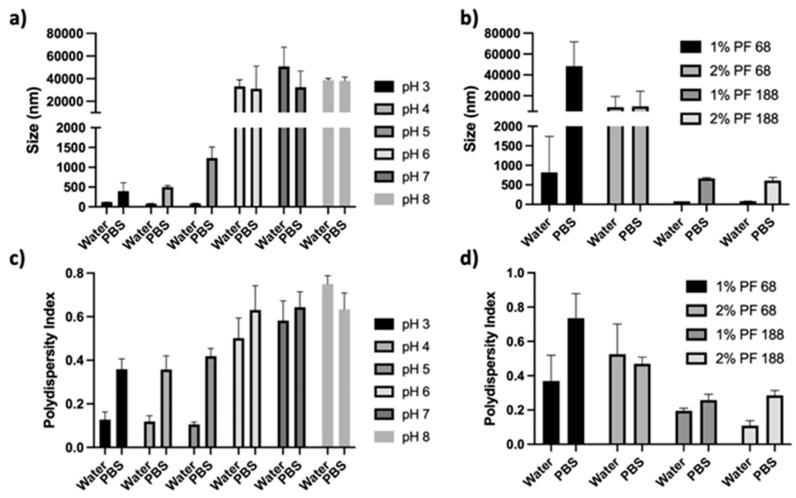
Post-functionalization of Zein nanoparticles (NPs) in varying experimental conditions: (**a**) size of Zein NPs post-functionalized with 3.0 mg/mL polyethylene glycol (PEG)-N-hydroxysuccinimide (NHS) in an unbuffered system adjusted directly with acetic acid; (**b**) size of Zein NPs post-functionalized with 3.0 mg/mL PEG-NHS in an unbuffered system containing 0.5% Tween 80 and varying levels of Pluronic (PF) 68 or 188; (**c**) polydispersity of Zein NPs post-functionalized with 3.0 mg/mL PEG-NHS in an unbuffered system adjusted directly with acetic acid; and (**d**) polydispersity of Zein NPs post-functionalized with 3.0 mg/mL PEG-NHS in an unbuffered system containing 0.5% Tween 80 and varying levels of PF 68 or 188.

PEG conjugation following the formation of Zein NPs (post-functionalized) was not successful (Figure 4 and Figure 5). Zein NPs were synthesized, and (i) the PEG-NHS ester concentrations (0.1, 1, 2, 3, and 4 mg/mL), (ii) the acid type (modulated using acetic acid or HCL), (iii) the temperature (room temperature 20 °C and 4 °C), (iv) the pH (3, 4, 5, 6, 7, and 8), (v) the presence of solvents (DMSO or EtOH), and (vi) the presence of stabilizers (Pluronic and Tween 80) were varied/considered. All samples where the concentration of PEG-NHS ester was varied showed NP aggregation. The lowest concentrations of PEG-NHS exhibited the smallest changes in size and PD (Figure 4a,b). [100:0] Zein:PEG-Zein NPs then had their pH lowered to a pH of 3 (from original pH of 6) using HCl, acetic acid, or citrate buffer. The citrate buffer was excluded from further analysis, as the particles demonstrated instability even before the PEG-NHS ester was added to the solution (Figure S1). While the NPs were stable in water with the addition of the PEG-NHS ester when using HCl or acetic acid to modulate the pH, it can be seen in Figure 4c,d that the Zein NPs aggregated in the presence of PBS. When solvents were added, 5% DMSO or 10% EtOH (Figure 4e–h, respectively), both the size and PD increased significantly in each formulation when exposed to PBS.

Temperature was decreased from room temperature (20 °C) to 4 °C to determine if the rate of aggregation could be decreased. The pH was adjusted using acetic acid to a pH of 3, 4, or 5. Additionally, samples were adjusted to a higher pH (a pH of 7 or 8) using NaOH directly, as weak bases could not be utilized due to the conjugation reaction used in the synthesis. As seen in Figure 5a,c, all formulations using these variations displayed an increase in size and PD when exposed to PBS. As anticipated, Zein NPs that were at their original pH (pH 6) or adjusted to higher pH levels near the protein’s isoelectric points aggregated when post-functionalized. The final synthesis parameter investigated was the addition of surfactants. In this case, all samples were synthesized as described in Section 2.3; however, the samples’ aqueous phases were modified with the addition of 0.5% Tween 80 and varying percentages of Pluronic (PF) 68 or 188 (Figure 5b,d) in a method described previously by our lab [18]. During the conjugation reaction, the solution was kept at 4 °C and the pH was not adjusted, while all other variables remained constant. It can be seen from Figure 5b,d that the formulations utilizing lower molecular weight PF formulations led to aggregation, even before the introduction of PBS. Higher molecular weight PF formulations, however, formed stable particles until PBS was introduced to the sample.

### 3.2. Zein-PEG Pre-Functionalization

**Figure 6:**
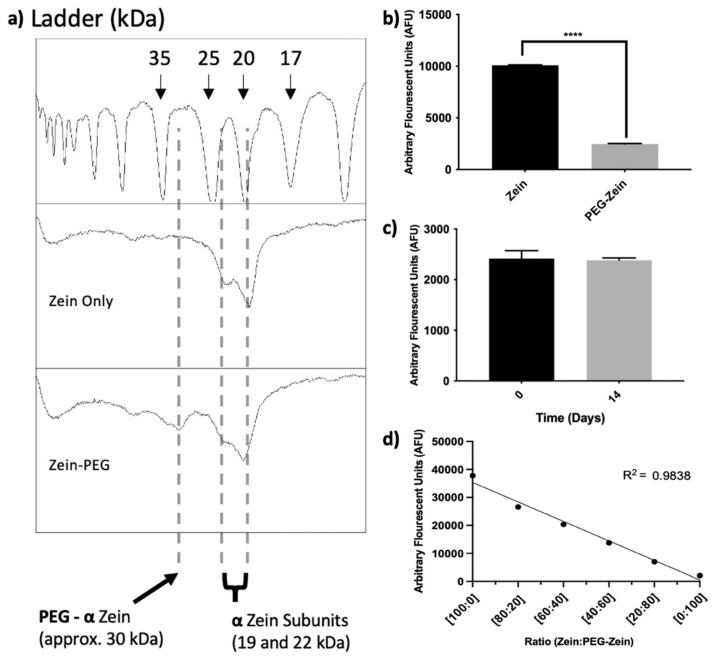
Pre-functionalization of polyethylene glycol (PEG) to Zein. (**a**) Densitometry of an SDS-PAGE displaying the emergence of a peak at 33 kDa, representing PEG-conjugated Zein; (**b**) fluorescent intensity of fluorescamine, reacting with the free primary amines on Zein, demonstrating a decrease in available primary amines once Zein is conjugated to PEG, (**c**) fluorescent intensity of fluorescamine for PEG-Zein at 0 and 14 days, representing the stability of the conjugation, and (**d**) fluorescent intensity of fluorescamine for Zein:PEG-Zein NPs at varying ratios of Zein:PEG-Zein. Statistical significance was declared at the following probability levels: **** *p* < 0.0001.

For pre-functionalized formulations, 1% purified Zein, which was solubilized in 60% EtOH, underwent a PEG conjugation reaction as described in Section 2.3. The conjugation of PEG to Zein was confirmed using two methods: (i) an SDS-PAGE gel (silver stain) and (ii) a fluorescamine assay which reacts to primary amines (the same function group used in for PEG conjugation). For the 22 and 19 kDa Zein subunits, a decrease in intensity was observed when the Zein sample was modified with PEG. A peak, at approximately 33 kDa, appeared in those samples, which underwent the conjugation reaction (Figure 6a). The fluorescamine assay demonstrated a four-fold decrease in fluorescence for Zein which had been modified with PEG (Figure 6b). This decrease was maintained over time as seen in Figure 6c. Optimization of the conjugation reaction varied the PEG-NHS concentrations, and it was determined that 8 mg/mL of PEG-NHS was sufficient to achieve similar levels of PEG modification as compared to a 16 mg/mL concentration of PEG-NHS (Figure S2).

It was important to demonstrate that the PEG-modified Zein could be used to form NPs and a final assessment measured fluorescamine binding to different Zein:PEG-Zein NPs synthesized using varying ratios of Zein (not PEG conjugated) to PEG-modified Zein. The results indicate that NP formation was not impacted by PEG modification and as expected (Figure 6d), the fluorescamine assay indicated that fluorescent labeling decreased as the PEG-Zein level increased in the formulations.

**Figure 7:**
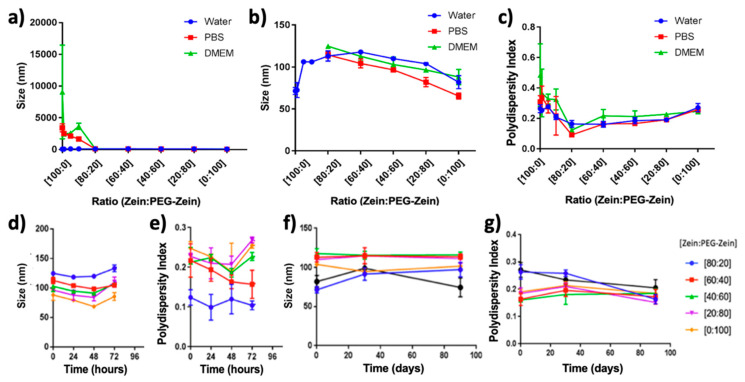
(**a**) Size as determined by dynamic light scattering (DLS) of [100:0], [99:1], [95:5], [90:10], [80:20], [60:40], [40:60], [20:80], and [0:100] Zein:PEG-Zein NPs in water, PBS, and DMEM after a 30 min incubation at room temperature, (**b**) size as determined by DLS of Figure 7a excluding [100:0], [99:1], [95:5], and [90:10] Zein:PEG-Zein NPs in PBS and DMEM, (**c**) polydispersity as determined by DLS of [100:0], [99:1], [95:5], [90:10], [80:20], [60:40], [40:60], [20:80], and [0:100] Zein:PEG-Zein NPs in water, PBS, and DMEM after 30 min incubation at room temperature; (**d**) size of the Zein:PEG-Zein NPs with varying ratios of Zein to PEG-Zein over time in DMEM at 37 °C; (**e**) polydispersity of the Zein:PEG-Zein NPs with varying ratios of Zein to PEG-Zein over time in DMEM at 37 °C; (**f**) size of the Zein:PEG-Zein NPs with varying ratios of Zein to PEG-Zein over time at 4 °C; and (**g**) polydispersity of the Zein:PEG-Zein NPs with varying ratios of Zein to PEG-Zein over time at 4 °C.

After confirmation of the PEG conjugation and NP formation, the NP formulations underwent further characterizations, including size, PD, (Figure 7a–c) and ζ potential (Figure S3). All Zein NPs synthesized with PEG-Zein exhibited a small increase in size (Figure 7a,b); the formulations had no statistically significant changes in PD when diluted in water (Figure 7c). Formulations with increasing ratios of PEG-Zein had only a minor decrease in their ζ potential (which ranged between 25 and 33 mV). The short-term stability of the various Zein NP formulations was assessed at 37 °C in DMEM. It can be observed that all formulations maintained the same size and PD for up to 72 h (Figure 7d,e). When assessing the formulations’ stability over three months at 4 °C, all formulations maintained their size and PD (Figure 7f,g). Formulations which were generated using a ratio above [80:20] Zein:PEG-Zein, e.g., containing less than 20% PEG–Zein, were identified as unstable in PBS and DMEM and, therefore, did not undergo further characterization (Figure 7a).

**Figure 8:**
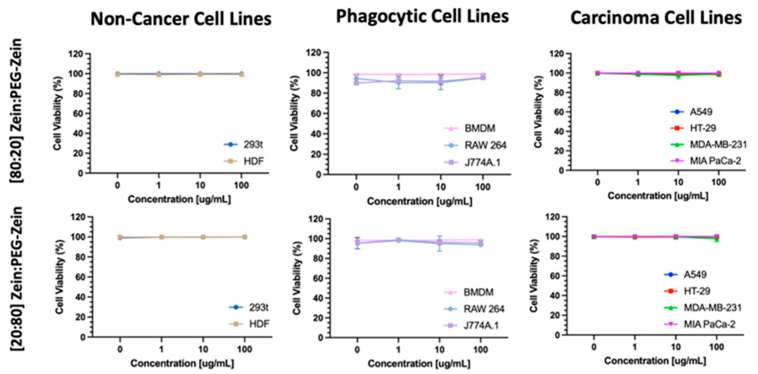
Cell viability at 72 h after [80:20] or [20:80] Zein:PEG-Zein NPs incubation at 0, 1, 10, or 100 µg/mL for non-cancer (293t and HDF), phagocytic (BMDM, RAW 264 and J774A.1) and carcinoma (A549, HT-29, MDA-MB-231, and MIA PaCa-2) cell lines.

High ([20:80] Zein:PEG-Zein) and low ([80:20] Zein:PEG-Zein) PEG-containing NP formulations were selected for in vitro studies to determine whether the formulations exhibited any cellular toxicity. The results, shown in Figure 8, demonstrate that there were no statistically significant changes in viability for non-cancer (293t and HDF), phagocytic (BMDM, RAW 264 and J774A.1), and carcinoma (A549, HT-29, MDA-MB-231, and MIA PaCa-2) cell lines when incubated with the NPs over a time of 72 h. Additionally, no increase in Caspase 3/7 activation was identified at 72 h in any of the cell lines (Figures S4–S8).

**Figure 9:**
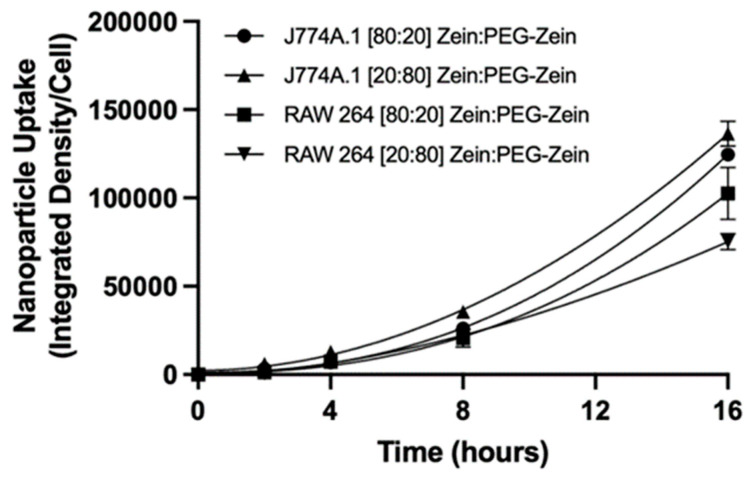
Phagocytic cell Zein nanoparticle (NP) uptake. RAW 264 and J774A.1 uptake over a 16 h period at 37 °C dosed with 1000 µg/mL of [20:80] or [80:20] Zein:PEG-Zein NPs coupled to CF-647.

It should be noted that there was little uptake of the Zein:PEG-Zein NPs ([80:20] and [20:80] Zein:PEG-Zein NP formulations) when uptake was assessed in the non-cancer and cancer cell lines, suggesting that there was an insignificant amount of NP endocytosis (data not shown). The phagocytic cell lines (J774A.1 and RAW 264) exhibited continuous NP uptake (Figure 9) over the 16 h time course (Figures S9 and S10). J774A.1 cells were identified as having a slightly higher rate of uptake for Zein:PEG-Zein NPs at 16 h relative to RAW 264 cells, an approximately 1.2 to 1.6 fold increase. It should be noted that DiI labeled DSPC:Chol (55:45) liposome containing no PEG, which displayed similar characteristics to the Zein NPs (e.g., near neutral charge with a size of 120 ± 15 nm), demonstrated significantly higher uptake at 2 h (Figure S11). Uptake was also found to be temperature dependent for both J774A.1 and RAW 264 cell lines (Figure S12).

**Figure 10:**
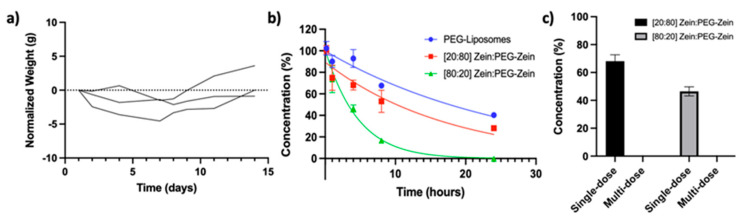
Safety and pharmacokinetic (PK) determination of Zein:PEG-Zein nanoparticles (NP)s: (**a**) two-week time course of weight changes in CD-1 mice dosed with 70 mg/kg of [80:20] Zein:PEG-Zein NPs; (**b**) PK profile of BALB/c mice dosed with 70 mg/kg of an [80:20] or [20:80] Zein:PEG-Zein NP formulation or with PEG-liposomes dosed at approximately 160 mg/kg of lipid; (**c**) concentration of [80:20] or [20:80] Zein:PEG-Zein NP formulations dosed at 70 mg/kg, either as a single exposure or after multiple exposures to the Zein NP formulations (e.g., the fourth and final dose of the multi-dose study).

In vivo safety studies were completed with the low ([80:20] Zein:PEG-Zein) PEG-containing NPs. The NPs were concentrated to as high a concentration feasible using TFF and sterile filtered before IV injection into CD-1 mice (Figures S13 and S14). The Zein:PEG-Zein NP dose was 70 mg/kg, and this was defined as the MFD for these studies. At this dose, the Zein:PEG-Zein NPs formulation displayed no signs of acute or long term toxicity. While there was a small decrease in weight (Figure 10a), the mice quickly returned to or surpassed their baseline weight. Overall, the NPs were well tolerated at the MFD. A second strain of immune-competent mice (Balb/c) were utilized to assess PKs following IV administration at the MFD and to assess whether the Zein:PEG-Zein NPs could be safely administered multiple times. For the single-dose PK, the [80:20] Zein:PEG-Zein NP formulation was eliminated from the plasma compartment more rapidly than the [20:80] Zein:PEG-Zein NP formulation (Figure 10b). Approximately 68% and 47% of the injected NP dose were observed in the plasma compartment after 4 h for the [20:80] and [80:20] Zein:PEG-Zein NPs formulations, respectively. To identify where the NPs accumulated, the mice had their organs imaged post-necropsy at the 4 h and 24 h timepoints. The imaging data indicated that the lungs and heart did not have detectable amounts of Zein NP present relative to background. On the other hand, the liver had a significant accumulation of Zein NPs regardless of the formulation or timepoint and, to a lesser extent, the kidneys, spleen, and lymph nodes displayed slightly higher fluorescent intensities relative to background (Figures S15 and S16).

To define whether the animals could be injected repeatedly with the PEG-modified Zein NPs, the animals were dosed every 7 days for the first three weeks with the last dose, dose 4, occurring at day 29. However, it should be noted that upon the administration of the second dose of Zein:PEG-Zein NPs, 50% of the mice had to be euthanized due to clinical signs of labored breathing. This included half of the group injected with [80:20] Zein:PEG-Zein NPs formulation and half of the group injected with the [20:80] Zein:PEG-Zein NPs formulation. Of the remaining animals, the NP formulations were given for 3rd and 4th injections of the multi-dose study. There were clinical signs of stress; however, they were not significant enough to warrant euthanasia. These clinical signs subsided within a few hours after administration. For those animals that did receive the NPs for three times, on the fourth injection, they were given the fluorescently tagged NP to allow for an assessment of plasma levels 4 h after the injection. These data could then be compared to the 4 h time point collected following a single dose PK scheme. The results (summarized in Figure 10c) demonstrated that no Zein NPs could be detected in the plasma if the animals were injected previously with Zein:PEG-Zein NPs. As indicated above for animals given a single IV dose of the NPs, there was approximately 68% and 47% of the injected dose in the plasma for animals given the [20:80] and [80:20] Zein:PEG-Zein NPs formulations, respectively.

## 4. Discussion

The goal of these studies was to prepare PEG-modified Zein NPs, as it was hoped that PEG modification would stabilize the NPs and would allow them to be developed for parenteral use, such as IV dosing. It is known that the PEG modification of lipid-based nanoparticles can prevent surface–surface interactions that lead to aggregation [17]. In this context, two strategies were developed to label liposomes with PEG. One approach was to add PEG-modified lipids to the lipid mixture prior to the formation of liposomes. The other took pre-formed liposomes, and PEG–lipid micelles were then mixed with those liposomes; over time, and pending conditions, the PEG–lipid would exchange into the outer leaflet of the liposome. The approaches considered for the Zein NPs were similar: modify preformed Zein NPs or modify Zein protein with PEG, and then incorporate the PEG-modified Zein protein into a NP. Many previous studies examined the coupling efficiencies of EDC/NHS and NHS esters for biological materials, such as proteins and nucleic acids [20,21,22,23]. In consideration of those studies, the approach described here to PEG modify preformed Zein NPs varied the ratio of NPs and the NHS ester, pH, temperature, and the presence of solvents. The results clearly demonstrate that regardless of conditions, pre-formed Zein NPs aggregate during the reaction. This is consistent evidence showing that pre-formed Zein NPs are not stable in the presence of physiologically relevant solutions. Thus, the studies focused on pre-conjugating the Zein protein with PEG prior to NP formation. It was a possibility that after PEG conjugation, the NPs would no longer form. However, this was not the case. By mixing PEG-conjugated Zein with unmodified Zein, NPs could be formed, and the resulting NPs were stable in the presence of salts and proteins if at least 20% of the Zein NP mixture included PEG-modified Zein.

To date, there has been only one publication detailing the post-functionalization of Zein NPs using PEG [24]. In this paper, steric stabilizers, such as lecithin and poloxamer, were utilized; however, details (including the concentration of each of the stabilizers, the pH, the percent of DMSO used, as well as the volume of the reaction mixture) were omitted, and the approach could not be reproduced here. All the results for the post-functionalization of Zein NPs highlight the importance of fully characterizing the system to promote reproducibility. It is possible that others will have better results with PEG modifying preformed Zein NPs than those described here. The pre-functionalization of Zein, however, appears to be a robust method to form Zein NPs that have associated PEG. EDC/NHS and NHS ester reactions were used previously to form folic acid conjugated Zein and PEG-Zein micelles [19,25,26,27,28,29]. Papers by Meewan et al. and Podarella et al. in particular characterize the chemical linkage of the PEG–NHS ester to Zein using NMR, FTIR, MALDI-TOF and SDS-PAGE and aligns well with our findings as well as those using similar N-terminus coupling methods, such as EDC/NHS [19,25,26,27,28,29]. While the majority of these papers investigate micelle-based formulations, it is likely that Zein micelles will be intrinsically unstable, disassembling into their subunits below their critical micelle concentration. This will limit the utility of Zein micelles in pharmaceutical applications [19,25,26,29].

Micelles are different from the ~100 nm Zein NPs described here. Stable Zein NPs were achieved and allowed the authors to consider future in vivo applications, such as for metastatic cancer, where IV dosing needs to be considered. The pre-functionalization of the Zein was achievable in 60% EtOH using an unbuffered system (Figure 5). Varying the concentration of PEG–NHS used to prepare the modified Zein protein demonstrated that for the Zein concentration, using a mole ratio of 1:3 of Zein to PEG–NHS was sufficient. In other words, having a greater excess of the PEG-NHS provided no further PEG conjugation of the Zein protein under the conditions used. This approach allowed the authors to capitalize on the inherent flexibility of the microfluidics process to control the Zein NP size while also allowing one to easily modulate the ratios of multiple conjugated components (e.g., PEG-Zein, dye-Zein, and prodrug-Zein) within the system. This system should also be suitable for use with bifunctional PEGs, allowing for other coupling reactions, such as biotin streptavidin, or for direct addition of a PEG terminated with a targeting moiety, such as folic acid. It is also interesting to speculate whether the PEG-modified Zein NPs described here could be used to further PEG-modify the PEG-Zein NPs with bifunctional PEGs after they have been formed. This may be a questionable idea, but the approach used here will have PEG molecules randomly oriented throughout the NP when the desired goal is to only modify the surface of the NP with PEG.

Zein NP uptake, transport, and cellular processing are still not well understood, in part because many Zein NPs prepared without PEG-modified Zein are unstable. The approach here allowed the authors to assess interactions between the Zein NPs and cells under conditions where the Zein NPs remained monodispersed. A study by Luo et al. described the uptake and transport of sodium caseinate (SC) coated Zein NPs [30]. In this paper, the formulation, which varies the ratio of SC to Zein, demonstrates no toxicity over the range of 200 to 1000 μg/mL. The lack of cellular toxicity of Zein NPs is consistent with other literature reports and the results described here (Figure 8) [31,32,33]. The study by Luo et al. identified a time, concentration, and temperature dependency on Zein NP uptake, which is in agreement with the findings in this paper [30]. Interestingly, Luo et al. indicated that their formulations’ mechanism of uptake was heavily reliant on clathrin- or caveolae-mediated endocytosis [30]. However, in a separate study by Dong et al. (who also used SC as a stabilizing agent for their Zein NPs), it was reported that the endocytosis uptake of their Zein NPs was not heavily reliant on caveolin- or clathrin-mediated pathways, but rather micropinocytosis [31]. These differences in findings may be due to several factors, including the different cell lines being utilized and their dependency on each pathway for NP uptake, the efficiency in pathway inhibition for each of the inhibitors used in the papers, the Zein NP formula differences, etc. [34]. While no Zein:PEG-Zein NP formulations have been assessed for cellular uptake in phagocytic cells, a study by Walkey et al. demonstrated that after a certain PEG grafting density was achieved on 90 nm gold NPs, there were no differences in phagocytic cell line uptake [35]. This finding is consistent with the studies here, where there was no significant difference between the uptake of the [80:20] and [20:80] Zein:PEG-Zein NPs into phagocytic cell lines (Figure 9). The presence of PEG can explain the decreased rate of uptake of the Zein:PEG-Zein NPs relative to the SC Zein NPs seen in Luo et al. and Dong et al., who already identified significant uptake in their formulations occurring within the scale of minutes to a few hours despite using similar concentrations of NPs as described in this paper [30,32,34,35,36,37]. A paper by Meewan et al. investigated the cellular uptake of Zein:PEG-Zein NPs in B16-F10-luc-G5 mouse melanoma cells with varying ratios of Zein:PEG-Zein and PEG chain lengths [38]. They identified a relative increase in Zein:PEG-Zein uptake when less PEG was utilized in the formulation containing 5 kDa PEG. This is contrary to the results presented here, which demonstrated no change in uptake. Possible explanations of this discrepancy may be due to the differences in experimental design, such as the cell type used, as well as the method of dye association. In the paper, coumarin-6 is passively associated with Zein:PEG-Zein NPs with varying levels of encapsulation efficiency for the different PEG containing formulations (with the lower PEG containing sample having an encapsulation efficiency 43% higher than the high PEG containing formulation). While the paper normalizes the total amount of coumarin-6 added to the cells for each of the formulations, it does not take into consideration the release characteristics of the different formulations that were generated [38]. In order to mitigate this potential, the paper presented here utilized a dye which was conjugated to the Zein via a physiologically stable amide bond. The conjugated dye would, therefore, not be subjected to potential differences in dissociation rates that could be seen in passively associated formulations. More research is still needed to better understand the influence of PEG on Zein NP uptake. Additional studies that focus on pathway-specific uptake mechanisms would be of interest, as it is known that PEG influences the cell binding and the uptake rate for other NP formulations [35,36].

One of the major challenges to be addressed here was the stability of Zein NPs. Instability limited the use of Zein NPs and previous in vivo studies used oral, intramuscular, subcutaneous, or intraperitoneal administration routes [39]. Formulas that did use an IV injection, often used a dose at or below a concentration of 1 mg/kg (almost 100-fold lower than the MFD used in these studies), typically only examine the immune response after a single dose [39,40]. While using a lower concentration would certainly increase the safety of the injection and may reduce formation of large aggregates after administration, the low concentration may limit the utility of such formulations as drug delivery systems. The data reported here suggest that PEG modification is an effective strategy to formulate highly stable particles, which demonstrate no acute or long-term toxicities. Despite Zein’s potential for use as a single-dose NP candidate, the PEG-modified Zein NPs could not be injected repeatedly. For those animals that were injected just two times, 7 days apart, there were significant repeat dose toxicities, severe enough to warrant euthanasia. It was expected that immune response to the Zein:PEG-Zein NPs would require weeks to develop; however it is postulated that the mice experienced significant toxicity after the second injection because the first dose resulted in generating an immunoglobin (Ig)M response. Many pathogens and foreign entities elicit an immune response that is characterized by an early elevation of antigen-specific IgM, followed by affinity maturation, isotype switching, and the subsequent increase in antigen-specific IgG, IgA and IgE antibodies [41]. Additional investigations should also be undertaken, using a variety of coatings of conjugated Zein NPs to ensure that the repeat dosing of conjugated PEG did not instigate the undesirable immune response reported here and by Ichihara et al. and others in non-Zein based PEG NP formulations [42,43,44]. Assuming that the toxicity was due to an immune response to the NPs, it is important to consider how Zein material is being used clinically. In clinical applications requiring sclerotherapy, Zein is administered locally as a viscous solution containing 210 mg of Zein, 162 mg of diatrizoate, 145 mg of oleum papaveris, and 6 mg of PEG per mL of EtOH. While no major complications were identified upon administration and long-term follow-up, minor, transitory complications, such as fever, local pain, and local inflammation to Ethibloc leakage in soft tissues, were identified [9,45]. Interestingly, the presence of fever, fever and pain, or fever, pain, and leakage was not correlated to the number of injected doses of Ethibloc [8,9]. Prophylactic therapy, including antibiotics, analgesics, and/or steroids, are commonly used to combat minor adverse reactions that take place upon Ethibloc injection [9]. This prophylactic modulation of the immune system preceding Ethibloc injection could be of interest for the NP formulation described here.

In addition to foreign proteins, there have been many well-documented adverse reactions to NPs made from a variety of materials which often limit their utility. Several of these reactions are linked to the interactions of the NPs with the immune system, including the activation of complement. This activation can cause well-characterized acute inflammatory reactions mediated by complement effectors and could ultimately be the cause of death in the Balb/c mice documented here. While non-PEG-modified Zein has been shown to increase IgG antibodies in Balb/c mice when injected intramuscularly, subcutaneously, and interpleural, more research is required to understand the immune response to the PEG-modified Zein NPs described here [39]. Will there be a way to mitigate the immune response raised against PEGylated Zein NPs upon multi-dose administration? The global or transient depletion of macrophages, such as by clodronate or chloroquine liposomes, could be utilized to change the immune responses to the PEG-modified Zein NPs [36,46]. At this time, the future use of these PEG-modified Zein NPs will be restricted to single-dose applications such as for gene therapies, hydroxocobalamin therapy for cyanide poisoning, or ceftriaxone therapy for syphilis [47,48].

## 5. Conclusions

The PEG modification of Zein NPs has been shown to be an effective method in the formation of stable NPs in physiologically relevant environments up to 72 h. These NPs could be synthesized to be approximately 100 nm with low PD (≤0.2 PD units) and near neutral charges (between 25 and 33 mV). Additionally, the NPs were shown to be stable at 3 months under storage conditions (4 °C) with no significant changes in their size or PD. Two methods of PEG conjugation were investigated: pre- and post- functionalization. The post-PEG functionalization of pre-formed NPs was assessed; however, under the conditions used here (varying the NHS concentration, pH, acid, presence of solvents, temperature, and presence of stabilizers), the modification reaction itself led to NP aggregation. The pre-functionalization of Zein prior to the formation of the NPs served as a robust method to form NPs with varying amounts of PEG. The amide bond formed using the PEG–NHS was shown to be stable at 14 days and allowed for easy modification between the ratios of Zein, PEG–Zein, and dye–Zein in the NP formulations. These Zein:PEG-Zein NPs were shown to be non-toxic to non-cancerous (293t and HDF), phagocytic (BMDM, RAW 264 and J774A.1), and carcinoma (A549, HT-29, MDA-MB-231, and MIA PaCa-2) cell lines with viability being ≥95% in all instances. Additionally, there was no identified increase in the apoptotic marker, Caspase 3/7, in any of the cell lines at 72 h. The NPs could be injected via an IV administration route at high doses (70 mg/kg) with no acute or long-term toxicities. Additionally, Zein:PEG-Zein formulations exhibited reasonable circulation longevities, with high PEG containing formulations retaining approximately 50% of its initial dose at 8 h and low PEG containing formulations at 4 h. While the approach is promising for single-dose applications, future studies probing the Zein:PEG-Zein NP immunogenicity for multi-dose applications are required.

## Data Availability

Data can be made available upon request.

## Supplementary Materials

The following supporting information can be downloaded at: https://www.mdpi.com/article/10.3390/pharmaceutics14091831/s1, Figure S1: Size and polydispersity of 2.5 mg/mL [100:0] Zein:PEG-Zein nanoparticles in various conditions; Figure S2: PEG-NHS pre-functionalization with varying concentrations of PEG-NHS; Figure S3: Characterization of Zein:PEG-Zein nanoparticles (NPs) for size, polydispersity, and zeta potential; Figure S4: 40× cell images of 293t and HDF cells incubated with various concentrations of [80:20] and [20:80] Zein:PEG-Zein nanoparticles and stained with Hoechst (DAPI) and CellEvent^®^ Caspase 3/7 (FITC) after a 72 h incubation; Figure S5; 40× cell images of RAW 264 and J774A.1 cells incubated with various concentrations of [80:20] and [20:80] Zein:PEG-Zein nanoparticles and stained with Hoechst (DAPI) and CellEvent^®^ Caspase 3/7 (FITC) after a 72 h incubation; Figure S6: 40× cell images of A549 and HT-29 cells incubated with various concentrations of [80:20] and [20:80] Zein:PEG-Zein nanoparticles and stained with Hoechst (DAPI) and CellEvent^®^ Caspase 3/7 (FITC) after a 72 h incubation; Figure S7: 40× cell images of MDA-MB-231 and MIA PaCa-2 cells incubated with various concentrations of [80:20] and [20:80] Zein:PEG-Zein nanoparticles and stained with Hoechst (DAPI) and CellEvent^®^ Caspase 3/7 (FITC) after a 72 h incubation; Figure S8: 40× cell images of bone marrow derived macrophage (BMDM) cells incubated with various concentrations of [80:20] and [20:80] Zein:PEG-Zein nanoparticles and stained with Hoechst (DAPI) and CellEvent^®^ Caspase 3/7 (FITC) after a 72 h incubation; Figure S9: 40× cell images of RAW 264 cells incubated with 1000 µg/mL of [80:20] Zein:PEG-Zein nanoparticles and stained with Hoechst (DAPI) over a 16 h time period; Figure S10: 40× cell images of J774A.1 cells incubated with 1000 µg/mL of [80:20] or [20:80] Zein:PEG-Zein nanoparticles and stained with Hoechst (DAPI) over a 16 h time period; Figure S11: 40× cell images of RAW 264 and J774A.1 cells incubated with DiI labled 120 nm polyethylene glycol (PEG) free liposomes (55:45 DSPC:Chol) or [20:80] Zein:PEG-Zein nanoparticles conjugated to CF-647 and incubated for 2 h at 37°C; Figure S12: 40× cell images of RAW 264 and J774A.1 cells incubated with 1000 µg/mL of [80:20] or [20:80] Zein:PEG-Zein nanoparticles and stained with Hoechst (DAPI) over a 16 h time period at 37°C and 4C; Figure S 13: Characteristics (concentration, size, and polydispersity) of [80:20] Zein:PEG-Zein nanoparticles post filtration varying filter material and filter pore size; Figure S14: Characteristics (size and polydispersity) of [20:80] Zein:PEG-Zein nanoparticles pre- and post- tangential flow filtration (TFF) varying speed; Figure S15: Organ distribution of Zein:PEG-Zein nanoparticles (NPs) at 24 h; Figure S16: Organ distribution of Zein:PEG-Zein nanoparticles (NPs) at 4 h.
