# Supplementary material for: PEG Conjugated Zein Nanoparticles for In Vivo Use
Source: Pharmaceutics. 2022 Aug 31;14(9):1831. doi: 10.3390/pharmaceutics14091831 (PMC9504474; doi:10.3390/pharmaceutics14091831)
Supplement: Supplementary file 1 [file pharmaceutics-14-01831-s001.zip › pharmaceutics-1826081-supplementary.pdf]

pharmaceutics

Article

PEG Conjugated Zein Nanoparticles for In Vivo Use

Table of Contents

Figure S1. Size and polydispersity of 2.5 mg/mL [100:0] Zein:PEG-Zein nanoparticles in various conditions..................2
Figure S2. PEG-NHS pre-functionalization with varying concentrations of PEG-NHS..2
Figure S3. Characterization of Zein:PEG-Zein nanoparticles (NPs) for size, polydispersity, and zeta potential 3
Figure S4. 40× cell images of 293t and HDF cells incubated with various concentrations of [80:20] and [20:80] Zein:PEG-Zein nanoparticles and stained with Hoechst (DAPI) and CellEvent® Caspase 3/7 (FITC) after a 72 h incubation..........4
Figure S5. 40× cell images of RAW 264 and J774A.1 cells incubated with various concentrations of [80:20] and [20:80] Zein:PEG-Zein nanoparticles and stained with Hoechst (DAPI) and CellEvent® Caspase 3/7 (FITC) after a 72 h incubation..........5
Figure S6. 40× cell images of A549 and HT-29 cells incubated with various concentrations of [80:20] and [20:80] Zein:PEG-Zein nanoparticles and stained with Hoechst (DAPI) and CellEvent® Caspase 3/7 (FITC) after a 72 h incubation..........6
Figure S7. 40× cell images of MDA-MB-231 and MIA PaCa-2 cells incubated with various concentrations of [80:20] and [20:80] Zein:PEG-Zein nanoparticles and stained with Hoechst (DAPI) and CellEvent® Caspase 3/7 (FITC) after a 72 h incubation..........7
Figure S8. 40× cell images of bone marrow derived macrophage (BMDM) cells incubated with various concentrations of [80:20] and [20:80] Zein:PEG-Zein nanoparticles and stained with Hoechst (DAPI) and CellEvent® Caspase 3/7 (FITC) after a 72 h incubation..........8
Figure S9. 40× cell images of RAW 264 cells incubated with 1000 μg/mL of [80:20] Zein:PEG-Zein nanoparticles and stained with Hoechst (DAPI) over a 16 h time period..........9
Figure S10. 40× cell images of J774A.1 cells incubated with 1000 μg/mL of [80:20] or [20:80] Zein:PEG-Zein nanoparticles and stained with Hoechst (DAPI) over a 16 h time period..........10
Figure S11. 40× cell images of RAW 264 and J774A.1 cells incubated with DiI labled 120 nm polyethylene glycol (PEG) free liposomes (55:45 DSPC:Chol) or [20:80] Zein:PEG-Zein nanoparticles conjugated to CF-647 and incubated for 2 h at 37°C..........11
Figure S12. 40× cell images of RAW 264 and J774A.1 cells incubated with 1000 μg/mL of [80:20] or [20:80] Zein:PEG-Zein nanoparticles and stained with Hoechst (DAPI) over a 16 h time period at 37°C and 4C..........12
Figure S13. Characteristics (concentration, size, and polydispersity) of [80:20] Zein:PEG-Zein nanoparticles post filtration varying filter material and filter pore size..........13
Figure S14. Characteristics (size and polydispersity) of [20:80] Zein:PEG-Zein nanoparticles pre- and post- tangential flow filtration (TFF) varying speed..........13
Figure S15. Organ distribution of Zein:PEG-Zein nanoparticles (NPs) at 24 h..........14
Figure S16. Organ distribution of Zein:PEG-Zein nanoparticles (NPs) at 4 h..........14

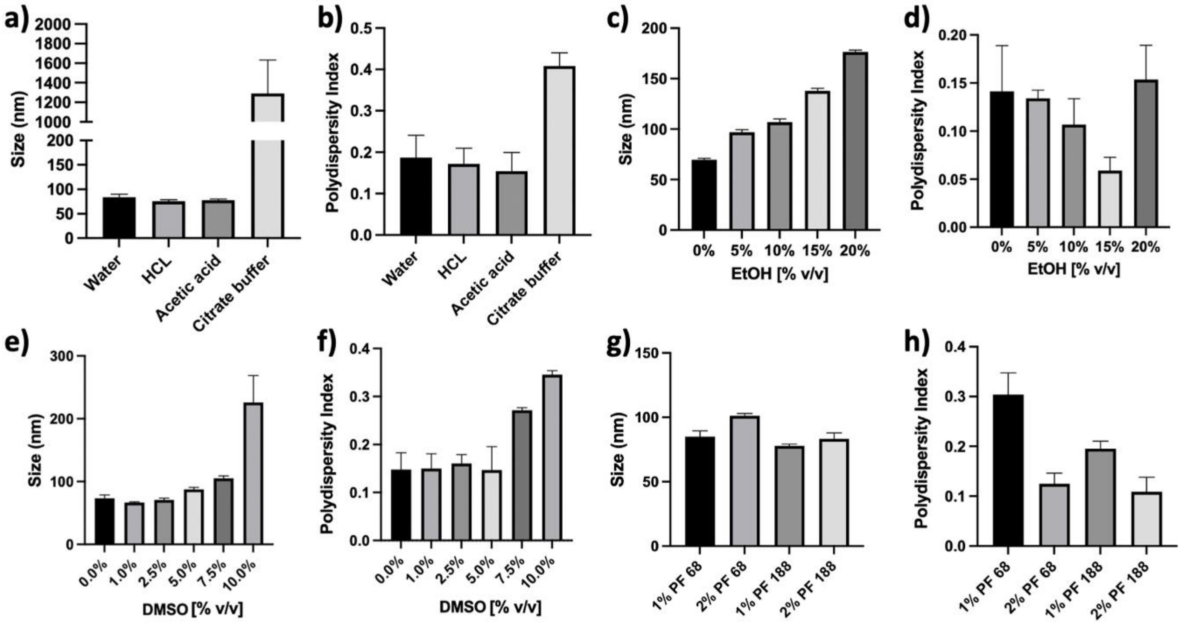

Figure S1: Size and polydispersity of 2.5 mg/mL [100:0] Zein:PEG-Zein nanoparticles in various conditions. (**a**) size of [100:0] Zein:PEG-Zein nanoparticles in unbuffered HCL and Acetic acid at pH 3 and citrate buffer at pH 3 after 24 h at room temperature, (**b**) polydispersity of [100:0] Zein:PEG-Zein nanoparticles in unbuffered HCL and Acetic acid at pH 3 and citrate buffer at pH 3 after 24 h at room temperature, (**c**) size of [100:0] Zein:PEG-Zein nanoparticles in various v/v % concentrations of EtOH after 24 h at room temperature, (**d**) polydispersity of [100:0] Zein:PEG-Zein nanoparticles in various v/v % concentrations of EtOH after 24 h at room temperature, (**e**) size of [100:0] Zein:PEG-Zein nanoparticles in various v/v % concentrations of DMSO after 24 h at room temperature, (**f**) polydispersity of [100:0] Zein:PEG-Zein nanoparticles in various v/v % concentrations of DMSO after 24 h at room temperature, (**g**) size of [100:0] Zein:PEG-Zein nanoparticles synthesized as described in section 2.3 with the variation of the aqueous phase containing both 0.5% Tween 80 and varying concentrations of Pluronic® (PF) 68 or 188, (**h**) polydispersity of [100:0] Zein:PEG-Zein nanoparticles synthesized as described in section 2.3 with the variation of the aqueous phase containing both 0.5% Tween 80 and varying concentrations of Pluronic (PF) 68 or 188. Synthesis protocol described in section 2.4.

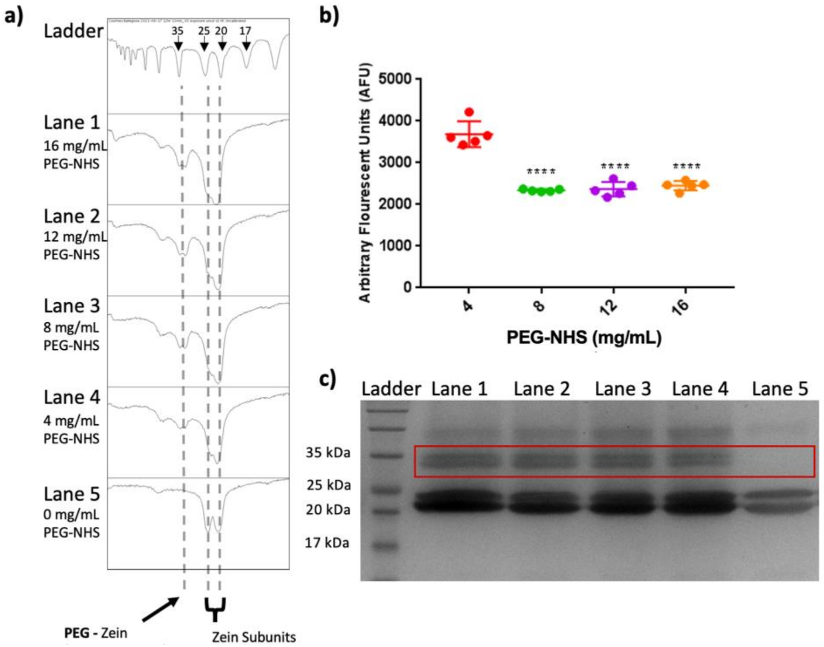

Figure S2: PEG-NHS pre-functionalization with varying concentrations of PEG-NHS. (**a**) densitometry of an SDS-PAGE displaying the emergence of a peak at 33 kDa, representing PEG conjugated Zein, when synthesized with varying concentrations of PEG-NHS while all other factors remain the same as described in Section 2.3., (**b**) fluorescamine assay, described in Section 2.6, of PEG-Zein conjugates synthesized with varying concentrations of PEG-NHS, (**c**) SDS-PAGE gel, as described in Section 2.5, of PEG-Zein conjugates synthesized with varying concentrations of PEG-NHS: lane 1 16 mg/mL of PEG-NHS, lane 2 12 mg/mL of PEG-NHS, lane 3 8

mg/mL of PEG-NHS, lane 4 4 mg/mL of PEG-NHS, and , lane 5 0 mg/mL of PEG-NHS. Conjugation protocol described in section 2.3. Statistical significance was declared at the following probability levels: **** $p < 0.0001$.

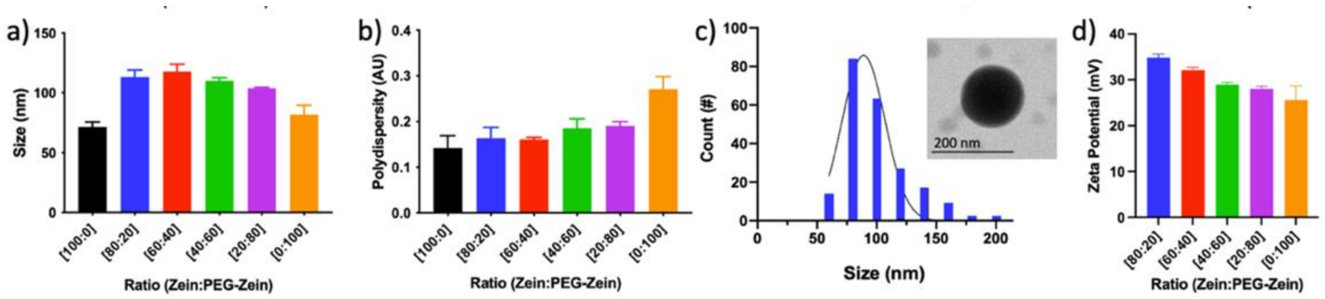

Figure S3. Characterization of Zein:PEG-Zein nanoparticles (NPs) for size, polydispersity, and zeta potential. (**a**) Size, as determined by dynamic light scattering (DLS), of the polyethylene glycol (PEG)-Zein nanoparticles (NPs) using microfluidics (total flow rate 3 mL/min; relative flow rate 3:1 aqueous to organic) with varying ratios of Zein to PEG-Zein; (**b**) Polydispersity of the Zein:PEG-Zein NPs with varying ratios of Zein to PEG-Zein, (**c**) transmission electron microscopy (TEM) of the [80:20] Zein:PEG-Zein NPs and size histogram derived from the TEM images; (**d**) ζ potential of the Zein:PEG-Zein NPs with varying ratios of Zein to PEG-Zein;

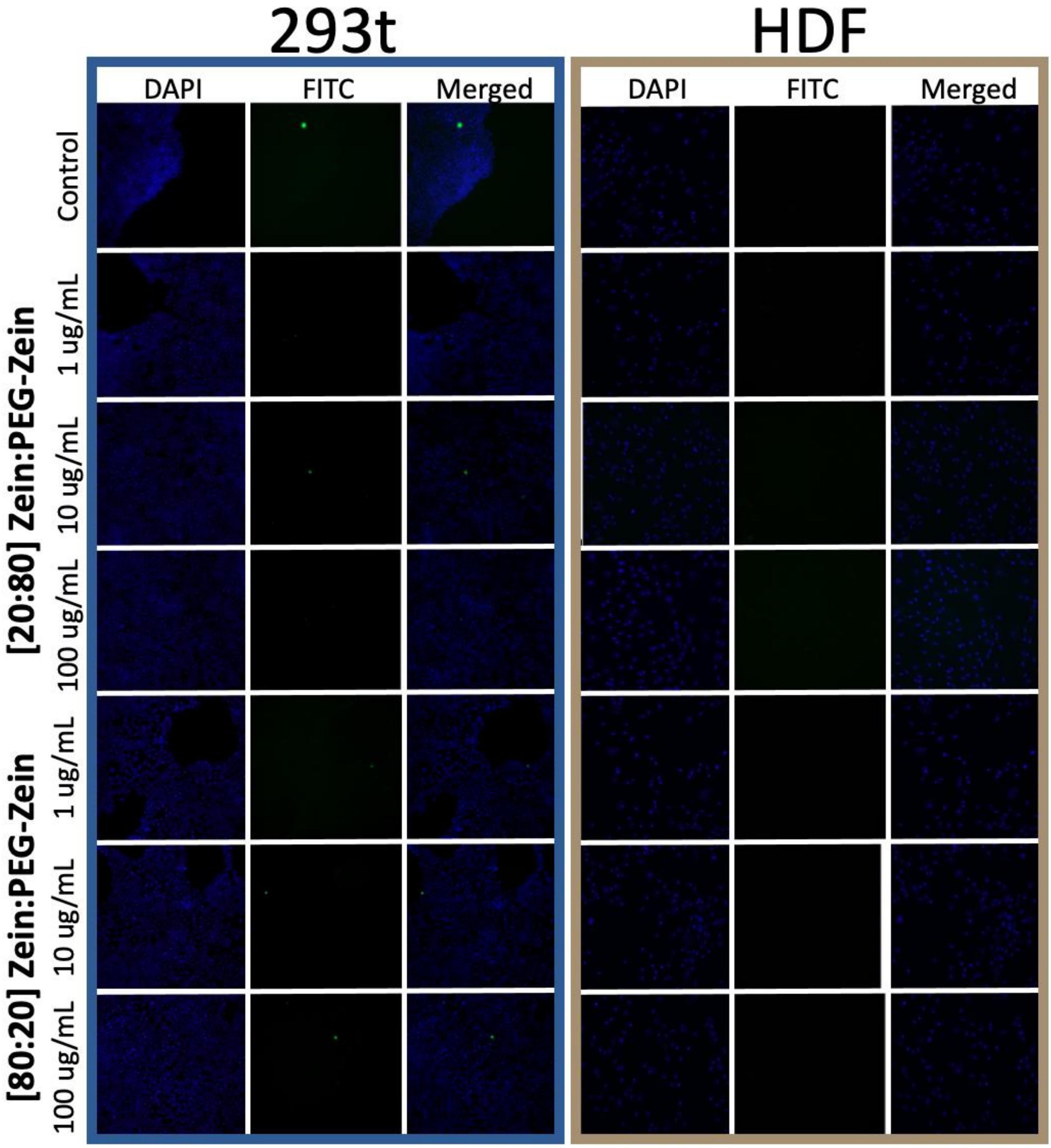

Figure S4: 40× cell images of 293t and HDF cells incubated with various concentrations of [80:20] and [20:80] Zein:PEG-Zein nanoparticles and stained with Hoechst (DAPI) and CellEvent® Caspase 3/7 (FITC) after a 72 h incubation. Full protocol described in Section 2.9.

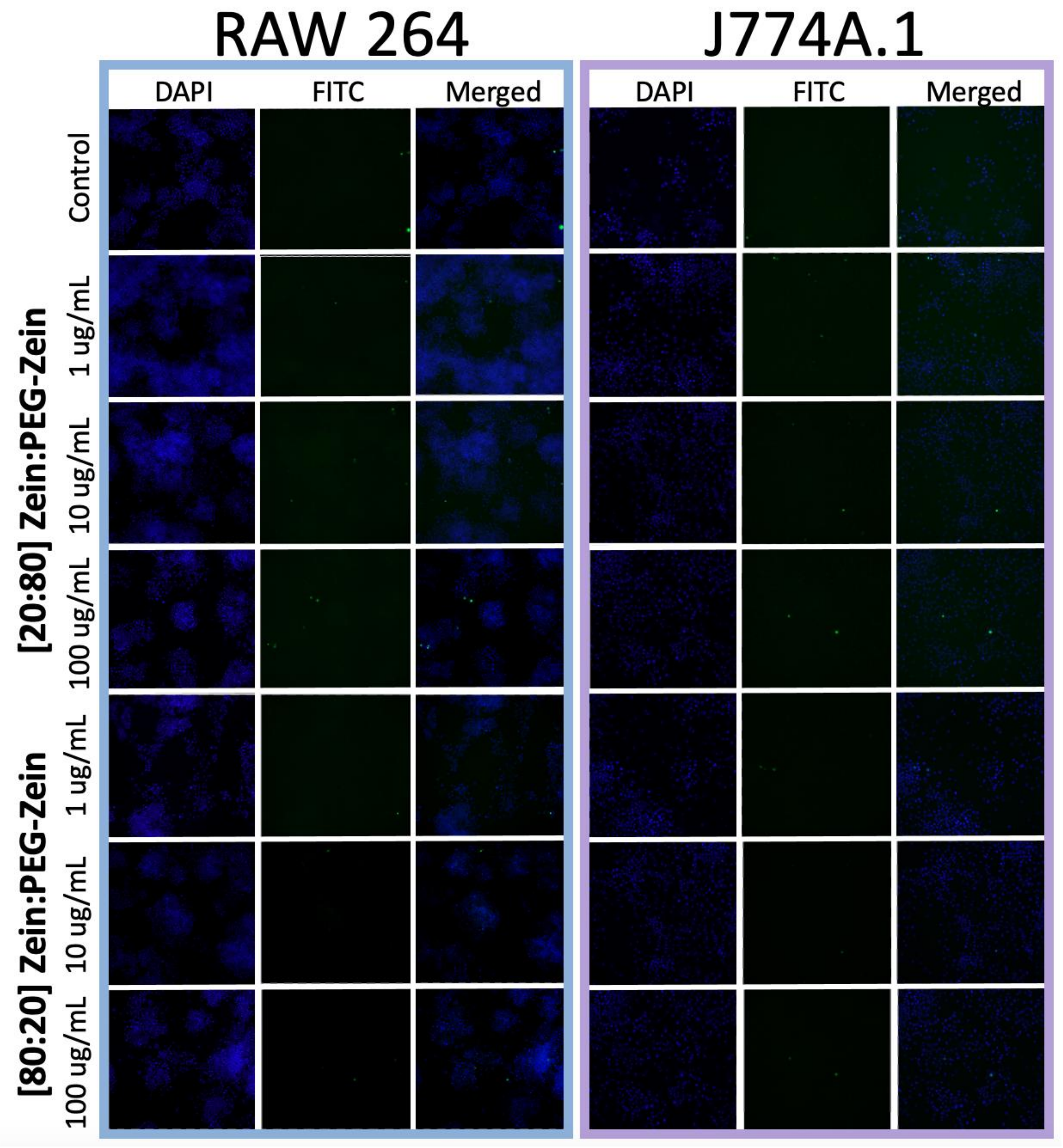

Figure S5: 40× cell images of RAW 264 and J774A.1 cells incubated with various concentrations of [80:20] and [20:80] Zein:PEG-Zein nanoparticles and stained with Hoechst (DAPI) and CellEvent® Caspase 3/7 (FITC) after a 72 h incubation. Full protocol described in Section 2.9.

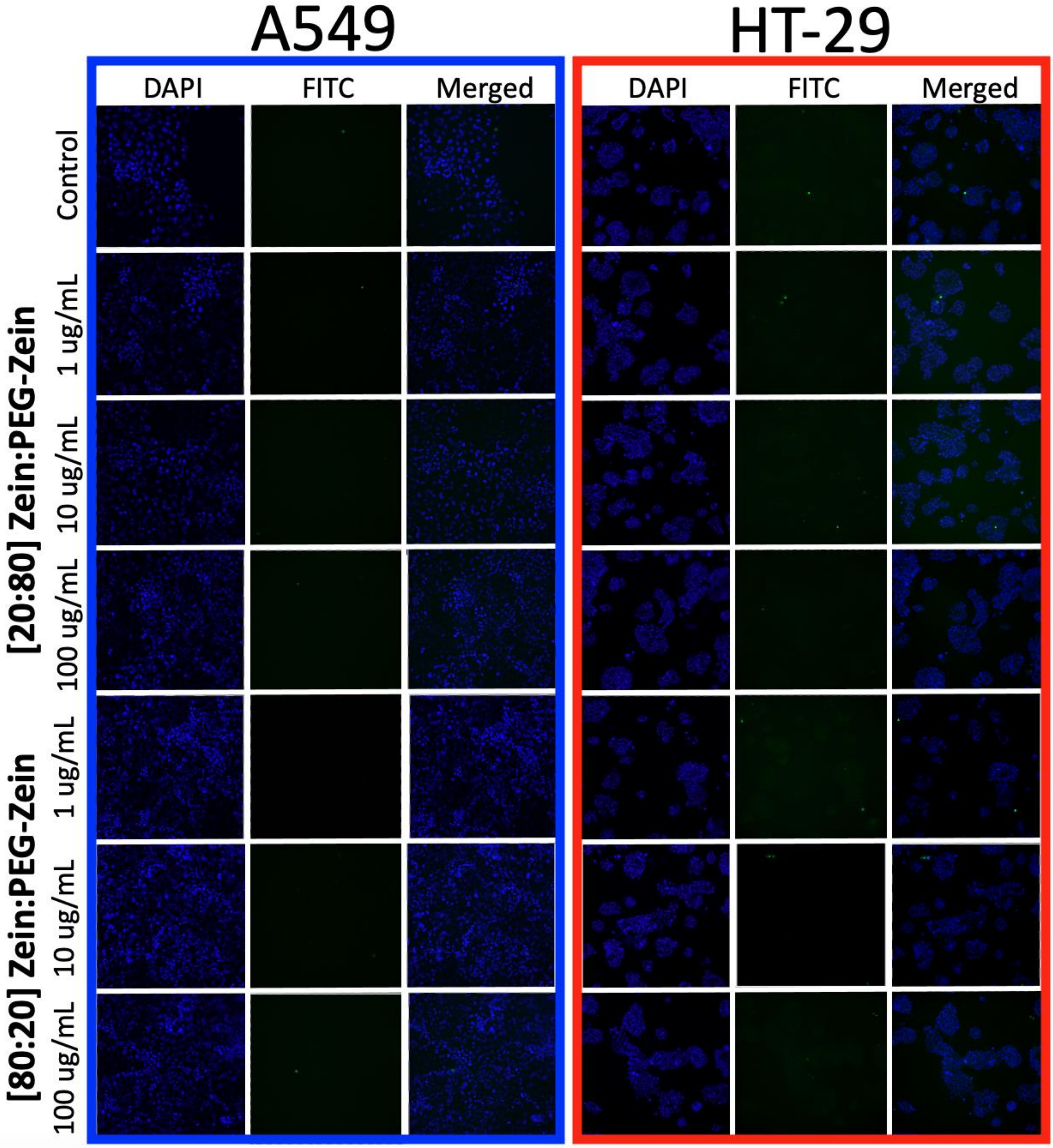

Figure S6: 40× cell images of A549 and HT-29 cells incubated with various concentrations of [80:20] and [20:80] Zein:PEG-Zein nanoparticles and stained with Hoechst (DAPI) and CellEvent® Caspase 3/7 (FITC) after a 72 h incubation. Full protocol described in Section 2.9.

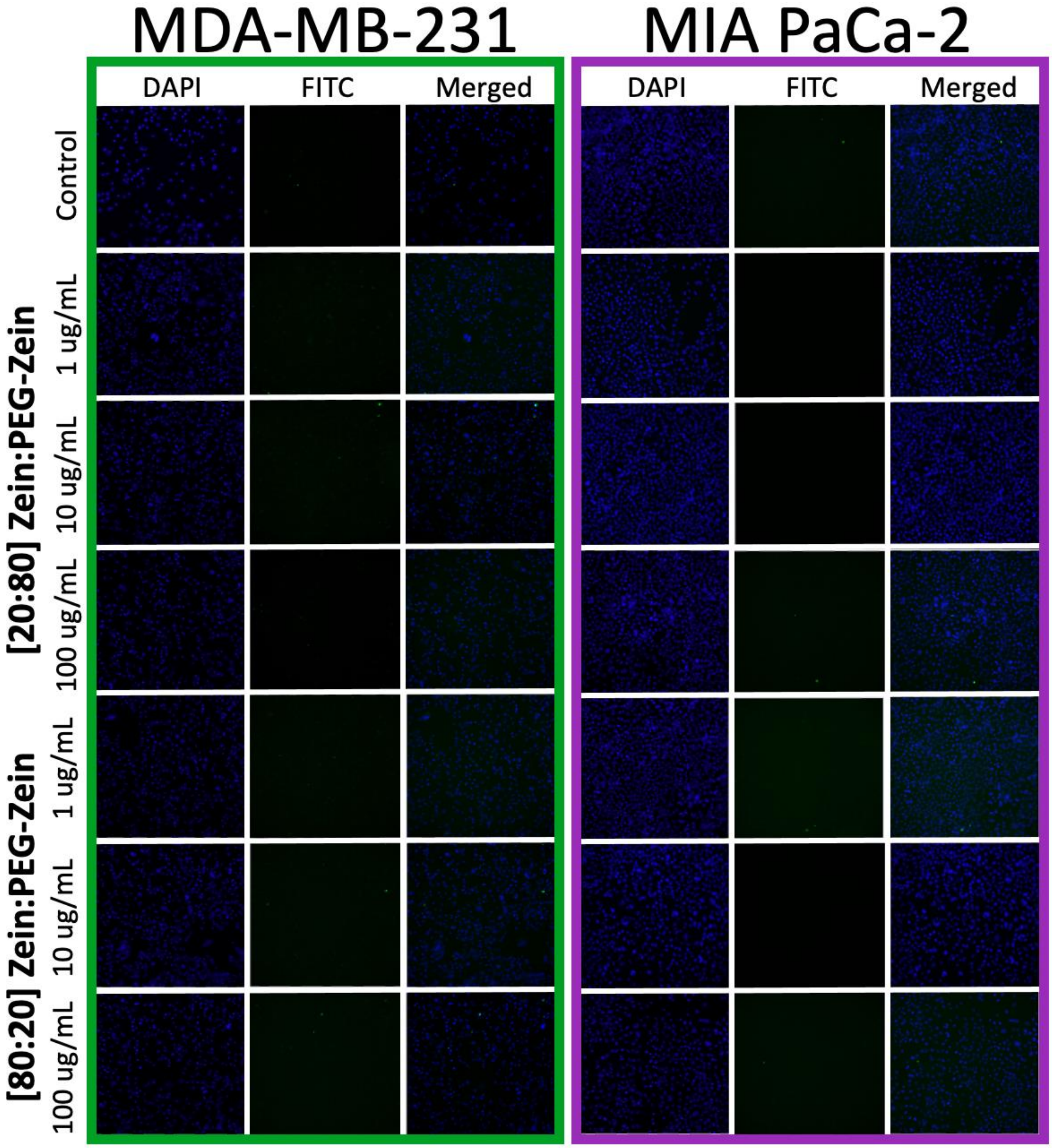

Figure S7: 40× cell images of MDA-MB-231 and MIA PaCa-2 cells incubated with various concentrations of [80:20] and [20:80] Zein:PEG-Zein nanoparticles and stained with Hoechst (DAPI) and CellEvent® Caspase 3/7 (FITC) after a 72 h incubation. Full protocol described in Section 2.9.

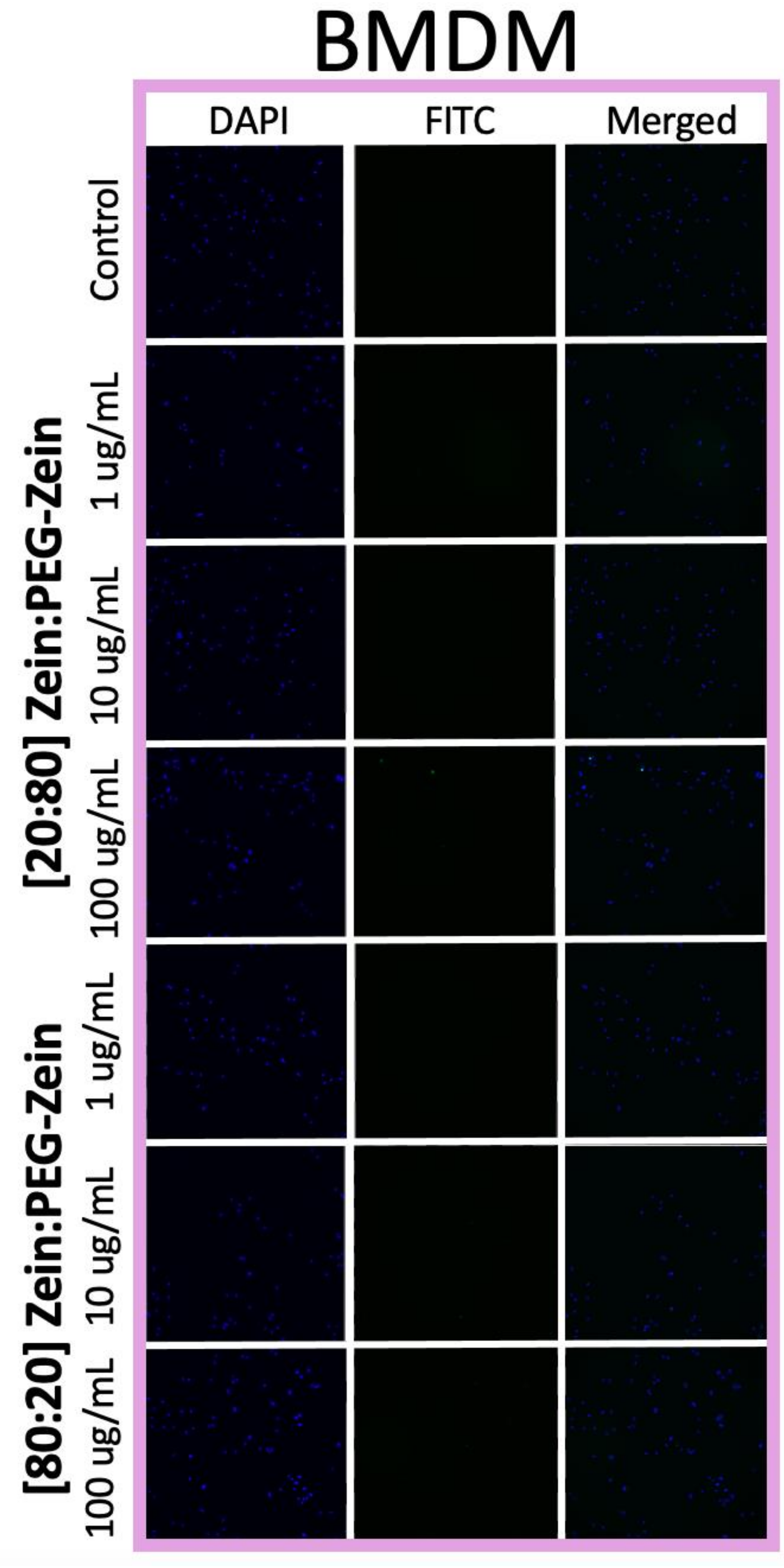

Figure S8: 40× cell images of bone marrow derived macrophage (BMDM) cells incubated with various concentrations of [80:20] and [20:80] Zein:PEG-Zein nanoparticles and stained with Hoechst (DAPI) and CellEvent® Caspase 3/7 (FITC) after a 72 h incubation. Full protocol described in Section 2.9.

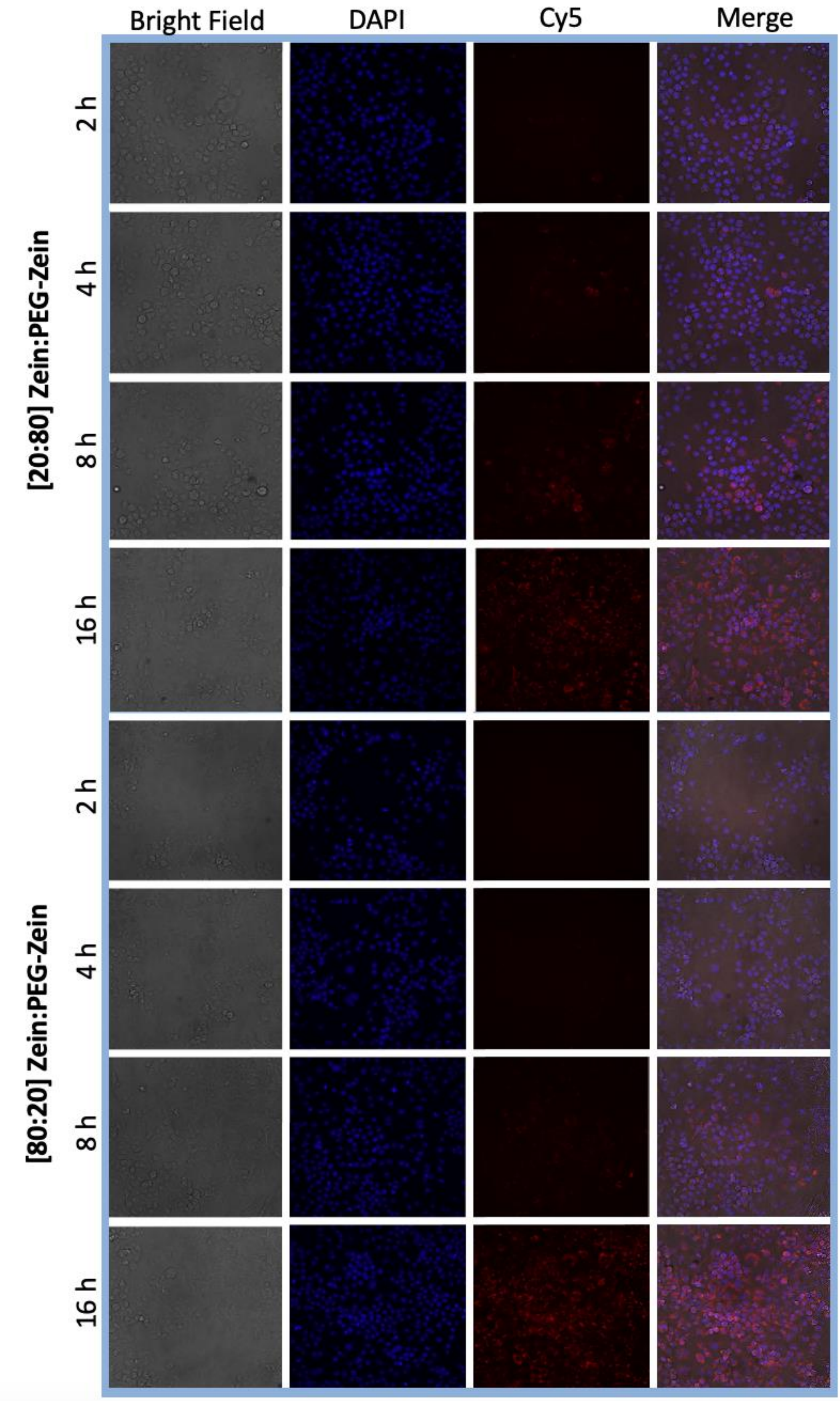

Figure S9: 40× cell images of RAW 264 cells incubated with 1000 μg/mL of [80:20] or [20:80] Zein:PEG-Zein nanoparticles and stained with Hoechst (DAPI) over a 16 h time period. Full protocol described in Section 2.10.

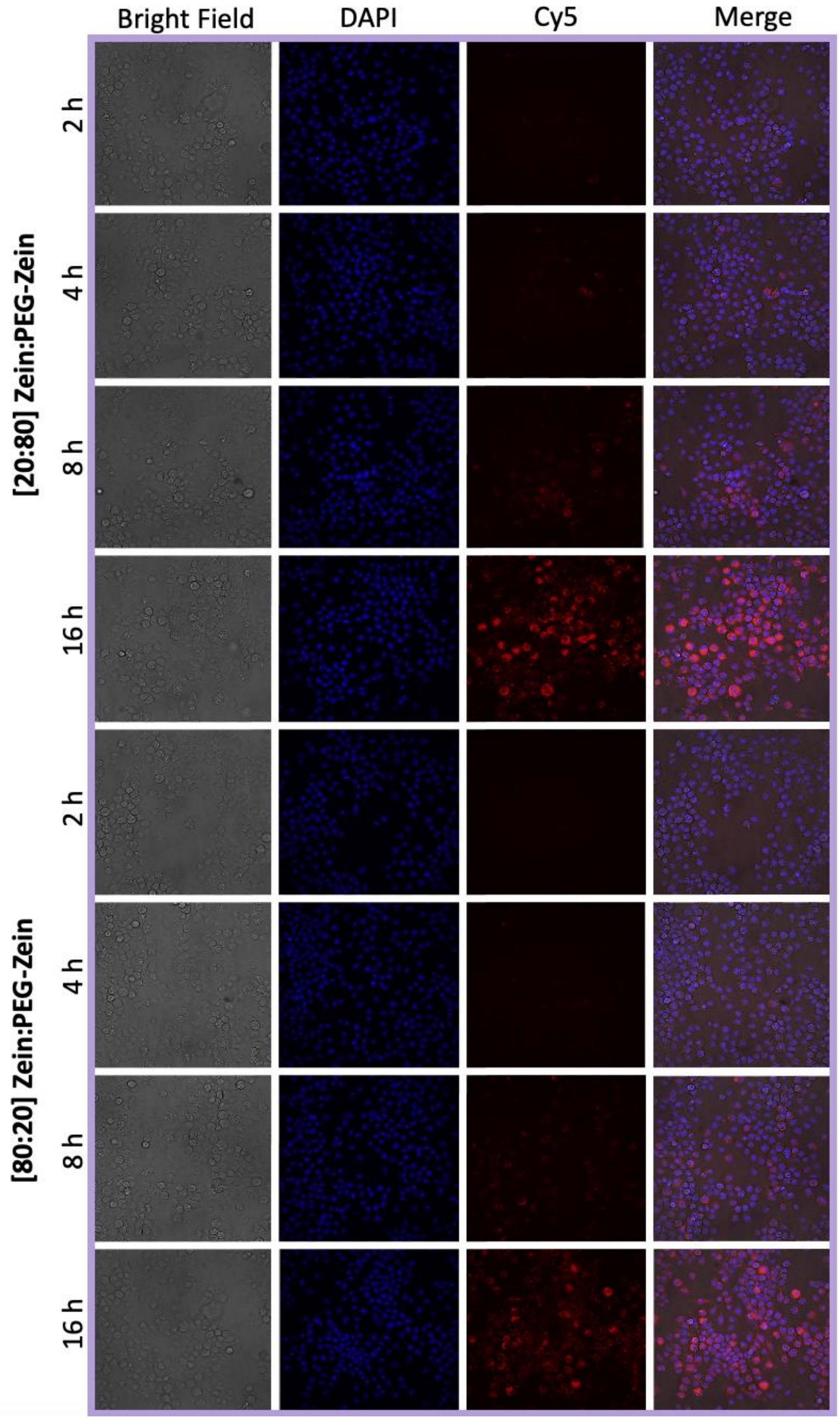

Figure S10: 40× cell images of J774A.1 cells incubated with 1000 μg/mL of [80:20] or [20:80] Zein:PEG-Zein nanoparticles and stained with Hoechst (DAPI) over a 16 h time period. Full protocol described in Section 2.10.

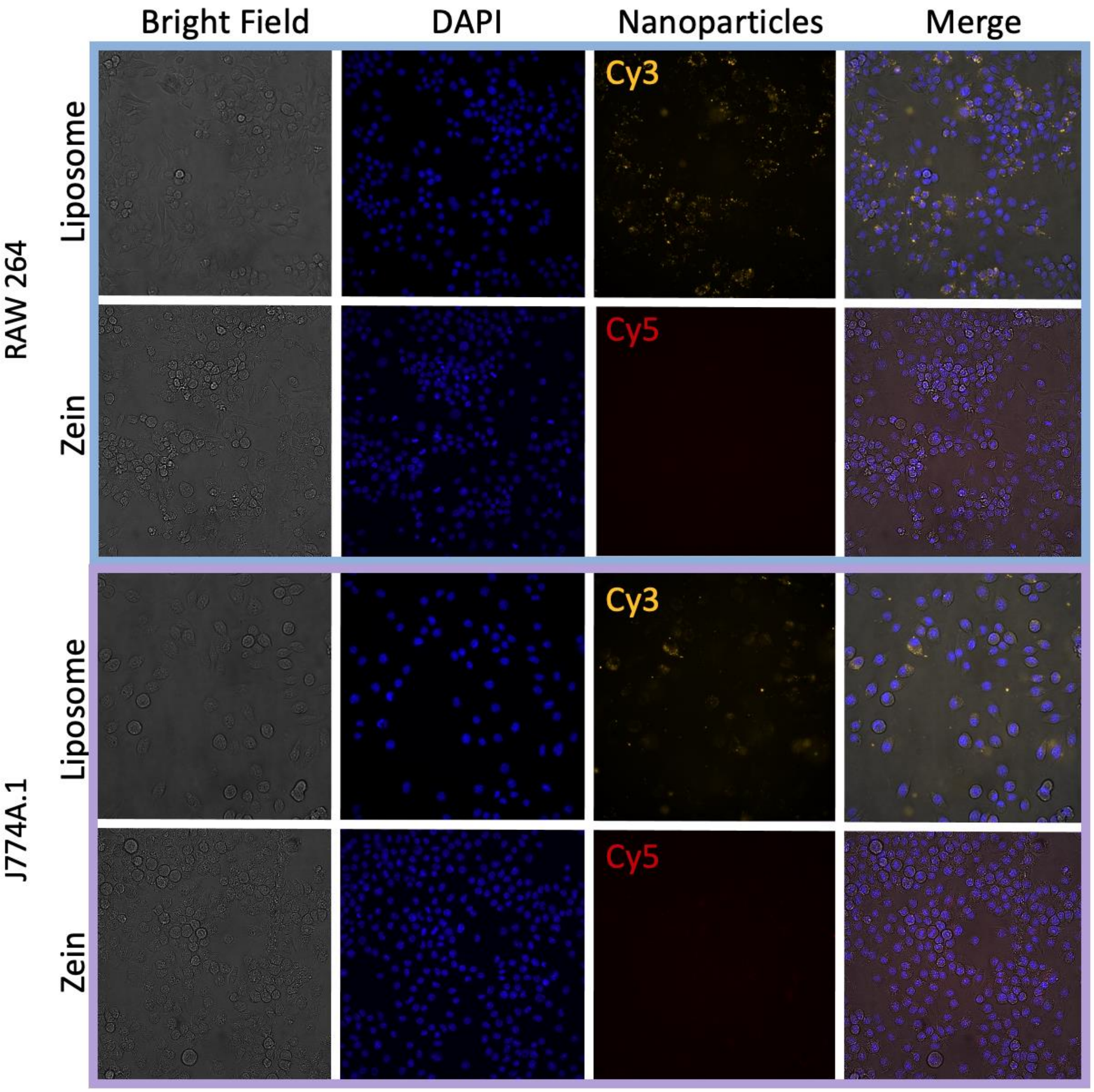

Figure S11: 40× cell images of RAW 264 and J774A.1 cells incubated with DiI labelled 120 nm polyethylene glycol (PEG) free liposomes (55:45 DSPC:Chol) or [20:80] Zein:PEG-Zein nanoparticles conjugated to CF-647 and incubated for 2 h at 37 °C. The uptake protocol in section 2.10 was followed for the experiment with a variation regarding the concentrations used for the nanoparticles. Both liposomes and Zein nanoparticles (NPs) were dosed with a final concentration of 5.08×10^{11} NPs/mL. A Mark-Houwink-Kakurada estimation for globular proteins of 120 nm (corresponding to a molecular weight of 42,407,510 g/mol) was utilized to determine the NP concentration of the Zein NPs assuming that the molecular weight of Zein is 20,500 g/mol.

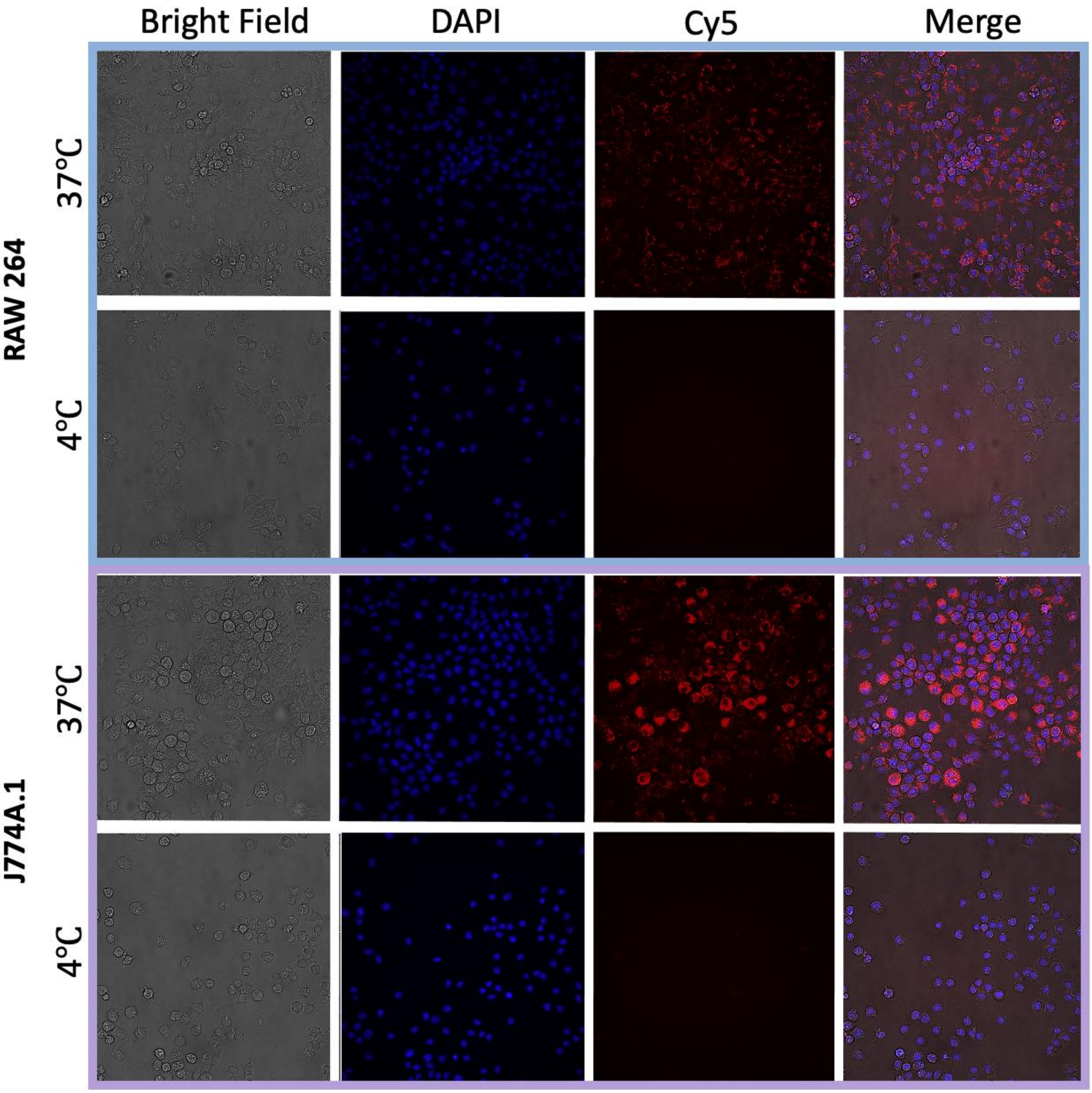

Figure S12: 40× cell images of RAW 264 and J774A.1 cells incubated with 1000 μg/mL of [80:20] or [20:80] Zein:PEG-Zein nanoparticles and stained with Hoechst (DAPI) over a 16 h time period at 37°C and 4C. Full protocol described in Section 2.10.

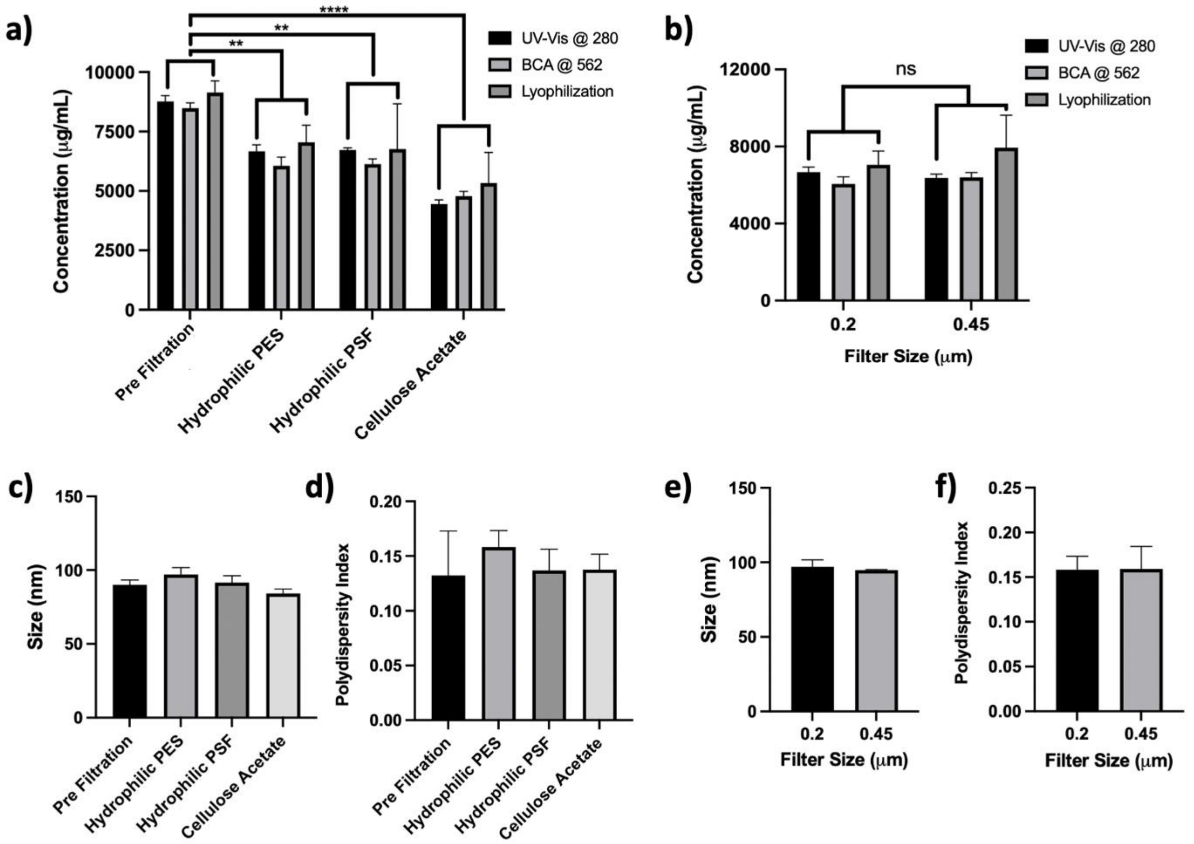

Figure S13: Characteristics (concentration, size, and polydispersity) of [80:20] Zein:PEG-Zein nanoparticles post filtration varying filter material and filter pore size. (**a**) concentration of Zein as determined by ultraviolet visible spectroscopy measured at 280 nm (UV-Vis @ 280), bicinchoninic acid assay (BCA) measured at 562 nm (BCA @ 562), and lyophilization pre- and post- filtration of [80:20] Zein:PEG-Zein nanoparticles varying filter material (0.2 μm filter pore size), (**b**) concentration of Zein as determined by UV-Vis @ 280, BCA @ 562, and lyophilization post- filtration of [80:20] Zein:PEG-Zein nanoparticles varying filter pore size (hydrophilic PES filter material), (**c**) size of material, as determined by dynamic light scattering (DLS), of samples in panel a, (**d**) polydispersity of material, as determined by DLS, of samples in panel a, (**e**) size of material, as determined by DLS, of samples in panel b, (**f**) polydispersity of material, as determined by DLS, of samples in panel b. Statistical significance was declared at the following probability levels: ** $p < 0.01$, and **** $p < 0.0001$.

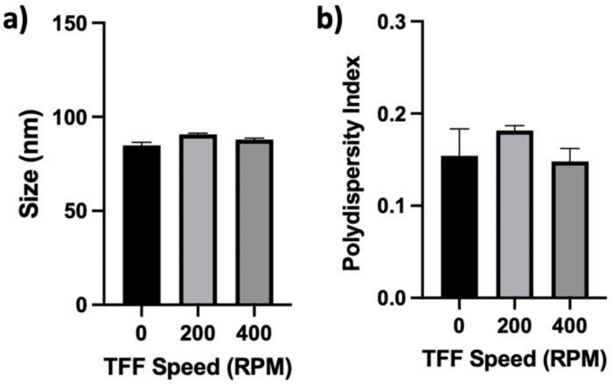

Figure S14: Characteristics (size and polydispersity) of [20:80] Zein:PEG-Zein nanoparticles pre- and post- tangential flow filtration (TFF) varying speed. (**a**) Size of [0:100] Zein:PEG-Zein nanoparticles pre- and post- tangential flow filtration (TFF) varying speed, (**b**) polydispersity of [20:80] Zein:PEG-Zein nanoparticles pre- and post- tangential flow filtration (TFF) varying speed.

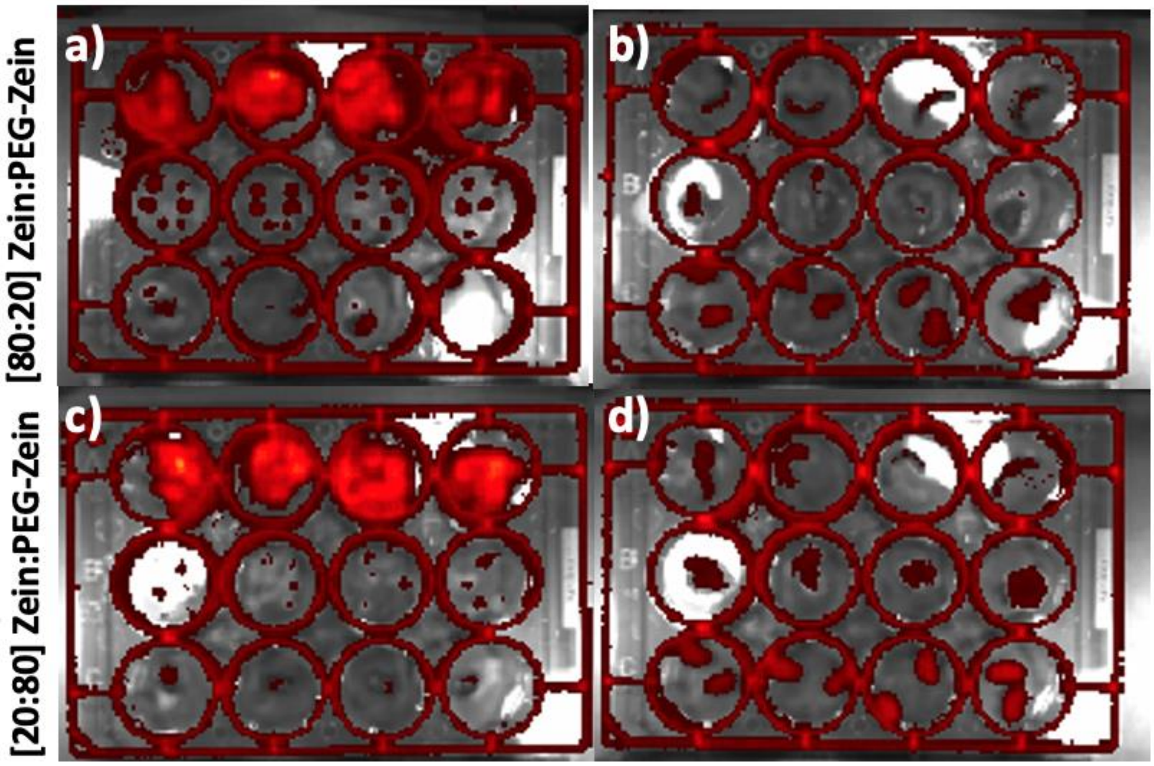

Figure S15: Organ distribution of Zein:PEG-Zein nanoparticles (NPs) at 24 h. (**a**) top row – liver (n = 4), middle row – lymph nodes (n = 4), and bottom row – heart (n = 4) post-necropsy 24 h after a single dose of [80:20] Zein:PEG-Zein, (**b**) top row – spleen (n = 4), middle row – lungs (n = 4), and bottom row – kidneys (n = 4) post-necropsy 24 h after a single dose of [80:20] Zein:PEG-Zein, (**c**) top row – liver (n = 4), middle row – lymph nodes (n = 4), and bottom row – heart (n = 4) post-necropsy 24 h after a single dose of [20:80] Zein:PEG-Zein, (**d**) top row – spleen (n = 4), middle row – lungs (n = 4), and bottom row – kidneys (n = 4) post-necropsy 24 h after a single dose of [20:80] Zein:PEG-Zein.

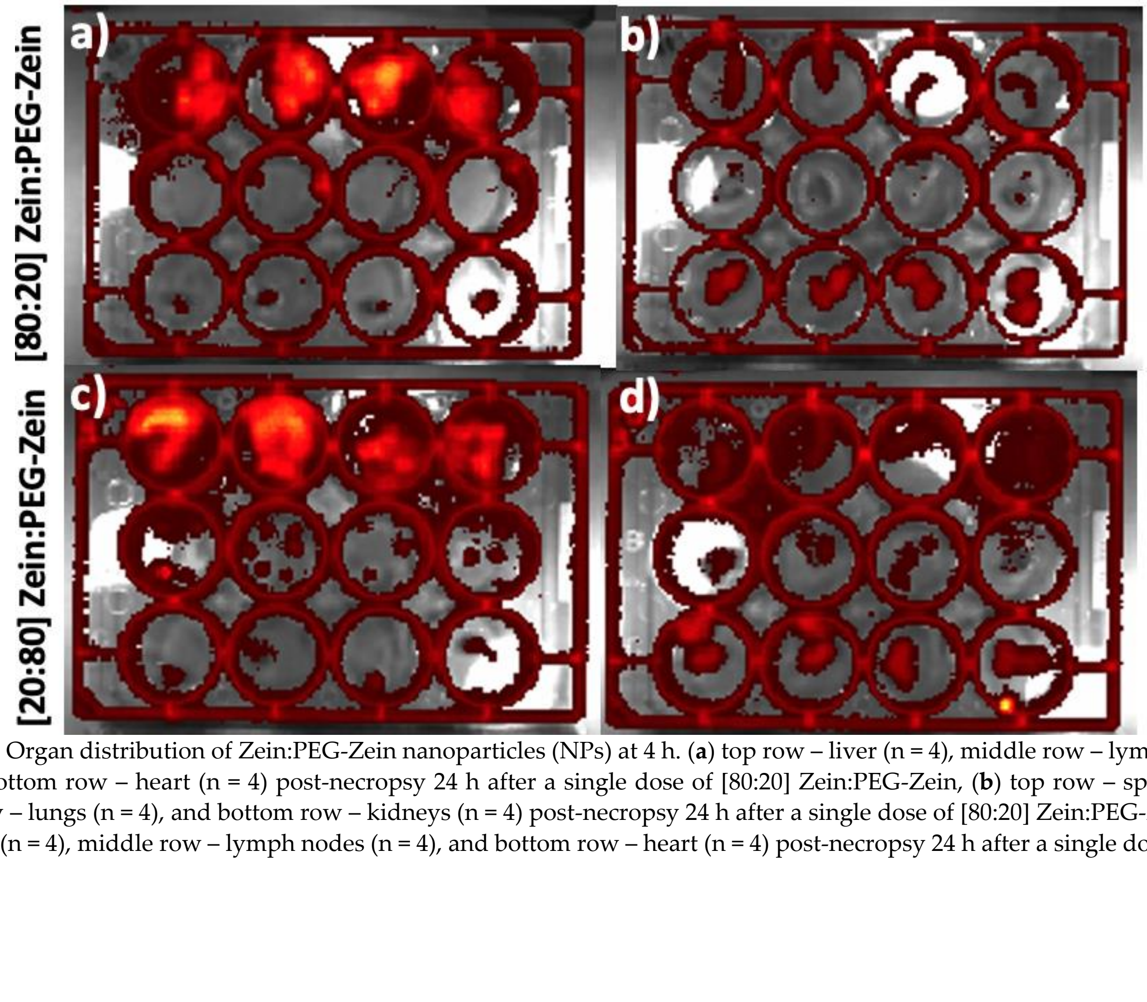

Figure S16: Organ distribution of Zein:PEG-Zein nanoparticles (NPs) at 4 h. (**a**) top row – liver (n = 4), middle row – lymph nodes (n = 4), and bottom row – heart (n = 4) post-necropsy 24 h after a single dose of [80:20] Zein:PEG-Zein, (**b**) top row – spleen (n = 4), middle row – lungs (n = 4), and bottom row – kidneys (n = 4) post-necropsy 24 h after a single dose of [80:20] Zein:PEG-Zein, (**c**) top row – liver (n = 4), middle row – lymph nodes (n = 4), and bottom row – heart (n = 4) post-necropsy 24 h after a single dose of [20:80]

Zein:PEG-Zein, (**d**) top row – spleen (n = 4), middle row – lungs (n = 4), and bottom row – kidneys (n = 4) post-necropsy 24 h after a single dose of [20:80] Zein:PEG-Zein.
